# The *E. coli* Anti-Sigma Factor Rsd: Studies on the Specificity and Regulation of Its Expression

**DOI:** 10.1371/journal.pone.0019235

**Published:** 2011-05-06

**Authors:** Nina Hofmann, Reinhild Wurm, Rolf Wagner

**Affiliations:** Institut für Physikalische Biologie, Heinrich-Heine-Universität Düsseldorf, Düsseldorf, Germany; Max-Planck-Institute for Terrestrial Microbiology, Germany

## Abstract

**Background:**

Among the seven different sigma factors in *E. coli* σ^70^ has the highest concentration and affinity for the core RNA polymerase. The *E. coli* protein Rsd is regarded as an anti-sigma factor, inhibiting σ^70^-dependent transcription at the onset of stationary growth. Although binding of Rsd to σ^70^ has been shown and numerous structural studies on Rsd have been performed the detailed mechanism of action is still unknown.

**Methodology/Principal Findings:**

We have performed studies to unravel the function and regulation of Rsd expression *in vitro* and *in vivo*. Cross-linking and affinity binding revealed that Rsd is able to interact with σ^70^, with the core enzyme of RNA polymerase and is able to form dimers in solution. Unexpectedly, we find that Rsd does also interact with σ^38^, the stationary phase-specific sigma factor. This interaction was further corroborated by gel retardation and footprinting studies with different promoter fragments and σ^38^- or σ^70^-containing RNA polymerase in presence of Rsd. Under competitive *in vitro* transcription conditions, in presence of both sigma factors, a selective inhibition of σ^70^-dependent transcription was prevailing, however. Analysis of *rsd* expression revealed that the nucleoid-associated proteins H-NS and FIS, StpA and LRP bind to the regulatory region of the *rsd* promoters. Furthermore, the major promoter P2 was shown to be down-regulated *in vivo* by RpoS, the stationary phase-specific sigma factor and the transcription factor DksA, while induction of the stringent control enhanced *rsd* promoter activity. Most notably, the *dam*-dependent methylation of a cluster of GATC sites turned out to be important for efficient *rsd* transcription.

**Conclusions/Significance:**

The results contribute to a better understanding of the intricate mechanism of Rsd-mediated sigma factor specificity changes during stationary phase.

## Introduction

Reprogramming the specificity of transcription during the change from exponential to stationary growth or under conditions of environmental stress is an essential feature of bacterial physiology. It requires that a large number of genes involved in growth and macromolecular synthesis are no longer expressed at high yield, while genes supporting maintenance and genetic stability, which are silent under exponential growth must be preferentially synthesized under conditions of generally shrinking resources [1]. Hence, the shift to stationary growth conditions is regulated by a complex network of cellular responses to reduce the now wasteful transcription of genes related to growth in favour to the expression of stationary phase-specific genes. This adaptive reaction is accomplished to a large extend by the use of alternative sigma factors. While transcription of the housekeeping genes under exponential growth is directed by σ^70^, the alternative σ factor, σ^s^ (σ^38^), is considered to be a master regulator for the adaptation to stationary phase transcription [2]. Cells have evolved a variety of mechanisms to support the alternative use of sigma factors responsible for the transcription of different regulons [3]. Individual σ factors can either be regulated by *de novo* synthesis on the transcriptional or translational level or on the activity of pre-existing factors. The regulation of synthesis often depends on the presence of small regulators, such as ppGpp or on mRNA stabilizing or destabilizing components. The stability or turnover of mRNAs for some σ factors is for instance affected by specific nucleases. In other cases inactive pre-sequences of σ factors are synthesized, which are activated by proteases, when needed. A common regulatory mechanism changing the activity of σ factors involves the action of proteins, which bind to σ factors, thereby inhibiting their association with the RNA polymerase core enzyme. These proteins are generally termed anti-sigma factors and notable examples are found for many different σ factors, including the housekeeping factor σ^70^ [4], [5].

Originally, the change in σ factor specificity between σ^70^, responsible for exponential growth, and σ^38^, responsible for stationary phase-specific transcription, was not easily comprehensible, because the concentration of σ^70^ exceeds that of σ^38^ at all growth phases. Moreover, the affinity of σ^70^ for core RNA polymerase is notably higher than that of σ^38^ [6], [7]. This apparent paradox has partly been solved by the discovery of cellular regulators, which reduce the activity of σ^70^, such as the non-coding 6S RNA, which selectively inhibits σ^70^ RNA polymerase holoenzyme (Eσ^70^) [8] and the characterization of the protein Rsd (regulator of sigma D), which is considered to act as an anti-sigma factor for σ^70^ [9]. The concentration of both regulators is significantly enhanced during the onset of stationary growth, consistent with their function in stationary phase adaptation [10], [11]. For both regulators, however, the σ^70^-specificity is not absolute [12], [13]. Although direct binding of Rsd to σ^70^ has been documented and analysed in detail, specific interaction was also shown to occur for instance between Rsd and the RNA polymerase core enzyme, suggesting that Rsd does not only sequester σ^70^ but might also affect the core enzyme of RNA polymerase [13]. Moreover, the fact that high concentrations of Rsd have been determined during exponential growth is difficult to reconcile with a simple mechanism of σ^70^ sequestering when cells enter stationary phase of growth [14]. The proposed function of Rsd as an anti-sigma factor might therefore involve more complex mechanisms as simply interfering with RNA polymerase holoenzyme formation by tight binding to σ^70^.

In order to elucidate details of the proposed anti-sigma factor mechanisms of Rsd, and to dissect the molecular steps, which ultimately lead to a shift in transcriptional specificity, we studied the potential interactions of Rsd with components of the transcription machinery.

For a complete understanding of the involvement of Rsd in stationary phase adaptation it is also important to learn more about the regulation of Rsd expression itself. Hence, we were interested to characterize details of the transcriptional regulation of the *rsd* gene to unravel its implication in stationary phase adaptation. Previous characterization of the *rsd* gene expression has demonstrated that its intracellular level increases during the transition from exponential growth to stationary phase [11]. The molecular details responsible for the growth phase-dependent expression are not known, however. We therefore characterized the influence of cellular effectors, known to be important for stationary phase expression, on transcription of the *rsd* gene *in vitro* and *in vivo.*

## Materials and Methods

### Bacterial Strains and Plasmids

The bacterial strains and plasmids used in this study are listed in Supplementary Table S1 and references are given in Supplementary References in Text S1.

### Isolation of proteins

The *E. coli* RNA polymerase core and holoenzyme (Eσ^70^) as well as the sigma factors σ^70^ and σ^38^ were isolated as described previously [15], [16], [17], [18], [19]. Native Rsd protein was isolated from BL21DE3pLysS/pUC18-Rsd cells grown in the presence of ampicillin. Protein expression was induced by IPTG (0.5 mM, 4 hours). Cells were lysed by sonication in the presence of 0.2 mM PMSF; 0.1 µM leupeptin and 0.1 µM pepstatin A. After ultracentrifugation the soluble protein fraction was separated on DEAE-Sephadex A25 followed by P11-phosphocellulose column chromatography. Rsd containing fractions were concentrated by NH_4_SO_4_ precipitation and stored in 10 mM Tris-HCl, pH 8.0; 200 mM NaCl; 10 mM MgCl_2_; 25% (v/v) glycerol; 0.2 mM EDTA, pH 8.0; 0.1 mM DTT; 0.2 mM PMSF; 0.1 µM leupeptin; 0.1 µM pepstatin A at −20°C. The DNA binding proteins H-NS, LRP, FIS and StpA were purified as described [10], [20], [21].

### Cross-Linking Studies

For cross-linking 3 µg Rsd protein was incubated for 30 minutes in a total volume of 15 µl 50 mM HEPES, pH 7.4; 5% (v/v) glycerol; 0.1 mM EDTA, pH 8.0; 0.1 mM DTT and 3 µg of either σ^70^, σ^38^ or Rsd, respectively. Samples were cooled on ice before 0.02% (v/v) glutaraldehyde was added. The reaction mixtures were then incubated at room temperature for another 30 minutes. The reaction was stopped by the addition of 5 µl 200 mM HEPES, pH 7.4; 20% (v/v) glycerol; 0.4 mM EDTA, pH 8.0; 0.4 mM DTT and 3 µl β-mercaptoethanol. Samples were denatured at 96°C for 90 seconds and analyzed on a SDS-gel. Protein bands were visualized by Coomassie staining.

### Biotinylation of Rsd

Rsd protein was dialysed against 50 mM HEPES, pH 7.4; 200 mM NaCl; 5% (v/v) glycerol; 0.2 mM PMSF for 2 hours at 4°C. A 9 fold excess of Biotinyl-N-hydroxy-succinimide was added to the reaction mixture in a total volume of 200 µl. Samples were incubated over night at 4°C. The biotinylation reaction was stopped by an excess of glycine and samples were dialysed against 50 mM HEPES, pH 7.4; 200 mM NaCl; 25% (v/v) glycerol; 0.2 mM PMSF and stored at −20°C. The efficiency of biotinylation was verified by SDS gel electrophoresis.

### Affinity Binding Assay

Biotinylated-Rsd protein was incubated with RNA polymerase core and holoenzyme at molar ratios of 1∶1 or 1∶3 for σ^70^, σ^38^ and native Rsd, respectively. Binding was allowed for 1 hour in 30 µl of TGED buffer (10 mM Tris-HCl, pH 8.0; 5% (v/v) glycerol; 0.1 mM EDTA, pH 8.0; 0.1 mM DTT) on ice. 100 µl of Streptavidine MicroBeads (Miltenyi Biotec) together with 70 µl TGED buffer were added and the mixture was incubated at room temperature for 5 minutes before it was placed on the column. The MicroBead column was washed four times with 100 µl TGED buffer to remove unspecific bound proteins. Specifically bound proteins were eluted with 150 µl of 50 mM Tris-HCl, pH 6.8; 50 mM DTT; 10% (v/v) glycerol; 1% (w/v) SDS. The flow-through and fractions from washing and elution were collected and precipitated with 4 fold volume of acetone for 2 h at −20°C. Precipitated samples were resuspended in 15 µl *Aqua dest.* and analyzed on SDS containing polyacrylamide gels. Protein bands were stained with Coomassie brilliant blue prior to silver staining.

### DNA-Fragments Used

The *rrnB* P1 fragment was obtained by cleavage of the plasmid pUC18-1 [22] with EcoRI and HincII. The fragment contains the coding strand (position −201 to +63, relative to the *rrnB* P1 start site). The *bolA*-fragment containing the coding strand (position −241 to +49, relative to the *bolA* P1 start site) was obtained by cleavage of pUC18-bolA with EcoRI and BsaAI. The *bolA* fragment for the analysis of the non-coding strand (position −160 to +120, relative to the P1 +1 start site) was obtained by cleavage of pUC18-bolA with HincII and XbaI. For modification with KMnO_4_ a DNA-fragment containing only the *bolA* P1 promoter (position -58 to 49 relative to the start site) was prepared by cleavage of pUC18-bolA1 with EcoRI and BsaAI. A 471 bp *rsd*-up-fragment, containing the *rsd* promoters and the upstream region, was obtained by cleavage of the vector pUC18-rsd-up with EcoRI and HincII. Additionally, a 288 bp long *rsd*-fragment containing the tandem-promoter *rsd* P1 and P2 was obtained by cleavage of the plasmid pUC18-rsd-up with BssHII and HincII. The 195 bp long up-fragment containing the upstream region without the *rsd* promoter was prepared by digestion of pUC18-rsd-up with the restriction enzymes SmaI and BssHII. A DNA fragment containing the *fic* promoter was generated by PCR from chromosomal DNA (*E. coli* K12 MG1655) with the primers: # fic2 5′-CTGGCCTGAAAATTACGAT-3′ and # fic-up 5′-GTTGCCGATAAGATTTCC-3′. The fragment was cleaved with BanI resulting in a 256 bp long fragment (position −157 to −99, relative to the +1 start site). Methylated DNA-fragments were derived from plasmids isolated from the *E. coli* strain XL-1 non-methylated plasmids were isolated from the *dam* deficient strain JM110, respectively. Methylation of the GATC-sites was verified by digestion with the methylation sensitive enzyme MboI. For binding and footprinting experiments DNA fragments were purified by agarose gel electrophoresis and end-labelled by Klenow (Promega) polymerase and incorporation of [α-^32^P]-dNTP.

### DNase I Footprinting

Limited DNase I cleavage of free and protein bound DNA was performed as described previously [23]. For the sequence specific A and G mapping chemical cleavage was performed as described before [24]. Bands were visualized by autoradiography. For quantitative analysis of the footprints the software *ImageJ 1.42q* was used.

### Modification of RNA Polymerase Complexes by KMnO_4_

Modification of DNA by KMnO_4_ was performed as described previously [25], [26]. Modified samples were cleaved by treatment with 10% (v/v) piperidine at 90°C for 30 minutes and cleavage products, after washing with *Aqua dest*. and lyophylization, were separated on 10% (w/v) denaturing polyacrylamid gels and visualized by autoradiography.

### Multiple Round *in vitro* Transcription

*In vitro* transcription reactions were performed in 50 mM Tris-acetate, pH 8.0; 10 mM Mg-acetate; 1 mM DTT; 0.1 mM EDTA, pH 8.0; 10 µg/ml BSA acetylated; 160 mM potassium-glutamate in the presence of 5 nM pSH666-1 plasmid together with 30 nM of each σ-factor, when indicated, and 65 µM ATP, GTP, UTP, 5 µM CTP and 133 nM [α-^32^P]-CTP. The reaction was initiated by addition of 20 nM holoenzyme together with the indicated Rsd concentration. New rounds of initiation were stopped after 10 minutes at 30°C by the addition of 6 µl chase-solution (1 mM Tris-HCl, pH 7.0; 2 mM ATP, CTP, GTP, UTP each, 2 µg heparin µl^−1^). After another 10 minutes at 30°C reactions were stopped with 10 µl stop-solution (250 mM EDTA, pH 8.0; 1 µl ^32^P-labelled DNA-fragment) and the samples were precipitated with ethanol, re-dissolved in 20 µl formamide-buffer and separated on denaturing polyacrylamide gels. Transcription products were visualized by autoradiography.

### Isolation of Total Cellular RNA

RNA was extracted from cells grown in YT-media at 37°C to either logarithmic or early stationary phase. Cells were cooled rapidly to 0°C and concentrated by centrifugation. Lysis and extraction of total RNA was performed as described previously [10]. RNA samples were routinely treated with RNase-free DNase I (Roche, Mannheim, Germany) and the quality of the preparation was verified by agarose gel electrophoresis.

### Primer Extension Analysis

For primer extension analysis 5 µg total RNA was incubated with 0.5 pmol 5′ [^32^P]-labelled desoxyoligonucleotide 5′-GCTCGGCGGATTTGTCCT-3′ complementary to position 160 to 142 of the *rsd*-mRNA generated by the vector prsd-up-cat. A second oligonucleotide 5′-TCAGCAGAGCGCAGATACCA-3′ complementary to position 28 to 9 of the RNA1 was used as an internal standard. Both oligonucleotides had been labelled at the 5′-end with [γ-^32^P] and T4-polynucleotidekinase (NEB, USA). The primer extension reaction was performed with the AMV reverse transcriptase (Promega, Madison, USA) as described previously [12]. Reaction products were separated on 15% (w/v) denaturing polyacrylamide gels and visualized by autoradiography. Product bands were quantified by using a phosphoimager (BioImager FAS 3000, Fuji, Japan).

## Results

### Interaction of Rsd with RNA Polymerase Studied by Glutaraldehyde Cross-Linking

While binding of Rsd to the σ^70^ subunit of RNA polymerase has been characterized intensively, the interaction with other RNA polymerase components is less clear. We therefore analyzed direct protein-protein interaction between Rsd and the different RNA polymerase sigma subunits σ^70^ and σ^38^ by glutaraldehyde cross-linking.

#### Rsd can be cross-linked to the specificity factors σ^70^ and σ^38^, *in vitro*

**Figure 1:**
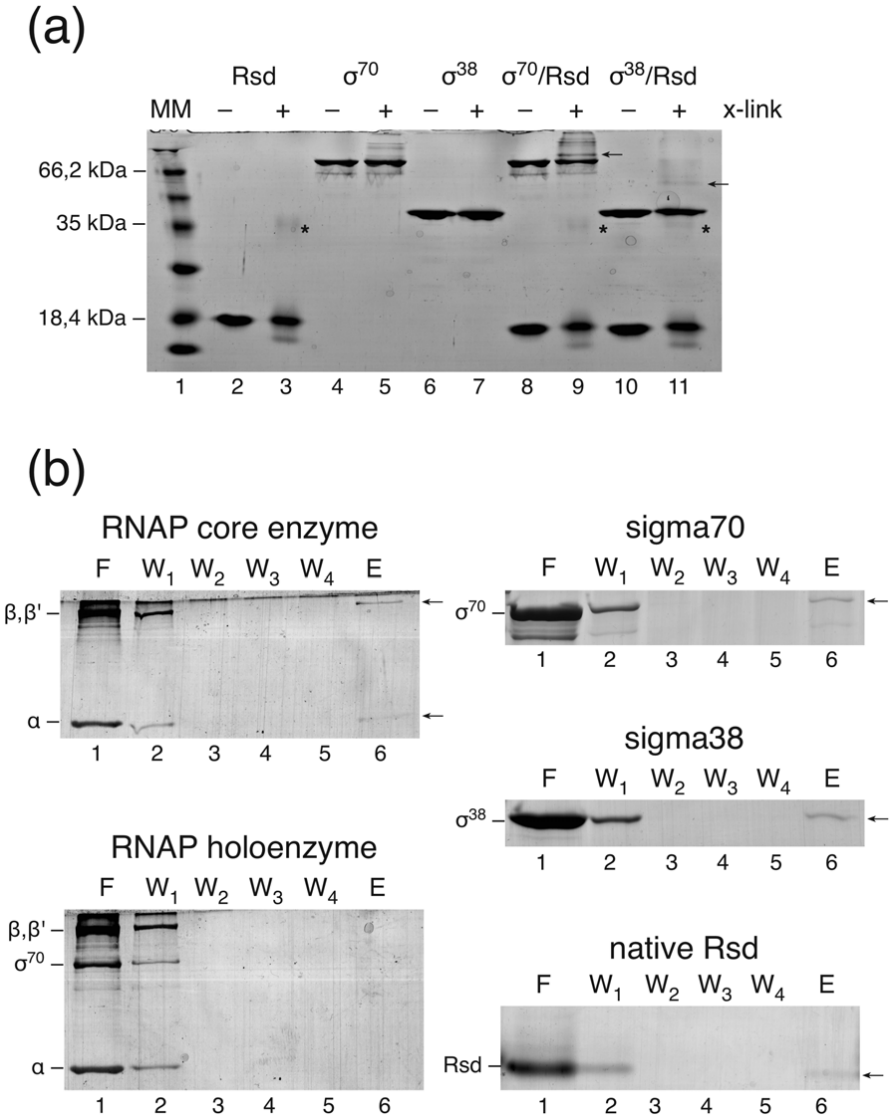
Direct interactions of Rsd with cellular proteins. (a) Cross-linking analysis of Rsd complexes with σ^70^ and σ^38^. Proteins (Rsd, σ^70^ and σ^38^, 3 µg each) were incubated in the presence or absence of the cross-linking reagent glutaraldehyde, either alone or together with Rsd, and separated on a denaturing SDS gel. The formation of possible homodimers was analyzed in lanes 3 (Rsd), 5 (σ^70^) and 7 (σ^38^). Heterologeous complex formation was analyzed in lane 9 (Rsd+σ^70^) and lane 11 (Rsd+σ^38^). The respective controls in the absence of cross-linker are shown in lanes 2 (Rsd), 4 (σ^70^) and 6 (σ^38^), 8 (Rsd+σ^70^) and 10 (Rsd+σ^38^), respectively. Lane 1 contains a molecular weight marker and characteristic size positions are given on the left margin. Heterodimes between Rsd and σ^70^ (lane 9) and Rsd and σ^38^ (lane 11) are indicated by arrows. Rsd dimers are indicated with an asterisk, the addition of cross-linker is marked with + and the lack of cross-linker with – above the lanes, respectively. (b) Affinity binding of Rsd to components of the *E. coli* transcription apparatus. Biotinylated Rsd was incubated with the protein of interest and the mixture was passed through columns of magnetic Streptavidin MicroBeads, which were fixed in a magnetic field. The columns were washed and eluted as given in Methods and the different fractions were separated on denaturing SDS gels. Lane 1 (F) indicates the flow-through. Lanes 2 to 5 represent consecutive washing fractions (W1 to W4). Lane 6 (E) shows the fractions eluted with SDS containing buffer to disintegrate potential protein complexes. Binding of Rsd was analyzed to RNA polymerase core enzyme α_2_ββ′ω (upper left panel), to RNA polymerase holoenzyme α_2_ββ′ωσ^70^ (lower left panel), to free σ^70^ (upper right panel), to free σ^38^ (middle panel on the right) and to Rsd itself (lower right panel), respectively. The positions of the individual proteins incubated with Rsd are given on the left margin of each panel. Arrows on the right mark bands indicative of bound proteins. Binding of Rsd could be observed for core RNA polymerase as well as to the isolated σ^70^ and σ^38^ subunits. Moreover, Rsd forms homodimers (or oligomers). For the RNA polymerase holoenzyme no protein could be eluted from the column (lane 6, lower left panel).

As a first approach to study possible interactions between Rsd and different components of the transcription apparatus glutaraldehyde cross-linking experiments were applied. Mixtures of the proteins of interest were incubated either alone or in the presence of Rsd with glutaraldehyde. An example of such an experiment is shown in Figure 1a. When the isolated proteins were analyzed we noted the tendency of Rsd to form dimers (marked by an asterisk on the gel presented in Figure 1a). Note that dimers have also been characterized for the related anti-sigma factor AsiA [27], [28]. No dimerisation or higher aggregate formation was visible for σ^38^ while for σ^70^ some aggregates can be observed following glutaraldehyde treatment. The existence of heterologous dimers between σ^70^ and Rsd are reflected by a cross-link band in the upper part of the gel (lane 9). While this interaction was expected the surprising formation of weak band(s) representing similar cross-link products between σ^38^ and Rsd is visible above the σ^38^ band in lane 11. In the case of σ^70^ and σ^38^ incubated together with Rsd and glutaraldehyde, the band intensities of free sigma factors and free Rsd are also noticeable reduced. We took this as a first indication that, in addition to the well established interaction of Rsd with σ^70^, the protein has also the potential to interact with σ^38^.

#### Characterization of potential Rsd interacting partners by affinity purification

Since cross-linking reactions always bear the risk of unspecific product formation we used an additional approach to verify the observed formation of Rsd-σ^38^ dimer complexes. As alternative method we performed affinity-binding assays employing magnetic beads and biotinylated Rsd (Figure 1b). The biotinylated Rsd was incubated with the different purified sigma factors, the mixture was subsequently attached to magnetic beads via streptavidin and used for column separation. After four consecutive washing steps specifically bound proteins were eluted with SDS containing buffer from the magnetic beads and separated by SDS gel electrophoresis. Results are exemplified in Figure 1b. The binding of Rsd to σ^70^ and also to σ^38^ is clearly confirmed by this method. Additionally, the homodimeric self-association of Rsd, as suggested by the cross-linking data, is corroborated by the affinity binding procedure (Figure 1b right panel).

We also investigated potential interactions between Rsd and RNA polymerase core (α_2_ββ′ω) and holoenzymes Eσ^70^ (α_2_ββ′ ωσ^70^). As can be seen in the upper left panel of Figure 1b Rsd is able to bind specifically to the RNA polymerase core enzyme, giving rise to core-specific proteins in the eluted fraction of the immobilized Rsd sample (lane 6, arrows pointing to β,β′ and α). This finding is in accordance with observations made by others [13]. However, when we tested binding of Rsd to the RNA polymerase holoenzyme (Eσ^70^), no protein that was retained from the affinity column could be detected (Figure 1b, lower left panel). Each experiment was performed in duplicate with identical results. The reliability of the method was further verified by non-binding control proteins, such as lysozyme or bovine serum albumin, which did not show any interaction (data not shown). Together, the results support the conclusion that at least *in vitro* Rsd does bind to the RNA polymerase core and both free sigma subunits, σ^70^ and σ^38^. However, Rsd does not interact with the holoenzyme of RNA polymerase. Furthermore, the results support the view presented previously that Rsd, like the well characterized T4 anti-sigma factor AsiA, might exist as a dimer [29], [30].

Several questions emerge from these observations. Binding of Rsd to σ^38^ appears to be at odds with the proposed function of Rsd as an anti-sigma factor, supposed to facilitate the switch from σ^70^ to σ^38^-dependent transcription. Moreover, binding of Rsd to the core enzyme, if it also occurs *in vivo*, suggests a more complex function as known for other well characterized anti-sigma factors [31].

#### Effects of Rsd on complex formation of σ^70^- and σ^38^-containing RNA polymerase holoenzymes with their corresponding promoters

The *Escherichia coli* protein Rsd is supposed to interfere with the specificity factor σ^70^ at the onset of stationary phase, *in vivo*. Rsd binding to σ^70^ will prevent the association with core RNA polymerase and formation of Eσ^70^ initiation complexes at σ^70^-specific promoters will be inhibited. As a consequence of the inactivation of σ^70^ the assembly of RNA polymerase holoenzymes with alternative σ factors, including the stationary phase-specific σ^38^-subunit, will be favoured [32], [33]. In order to better understand the effects of Rsd as transcriptional regulator and to elucidate how Rsd contributes to the switch in the transcriptional adaptation of cells, which undergo transition between exponential and stationary growth we initiated binding and footprinting studies of Rsd in combination with different RNA polymerase-promoter complexes. Lead by the surprising observation that Rsd is apparently able to interact with σ^38^ we compared the effect of Rsd on specific RNA polymerase holoenzymes Eσ^70^ and Eσ^38^ bound to their cognate promoters (*rrnB* P1 and *bolA*, respectively).

#### Rsd interferes with the ability of RNA polymerase to form functionally active complexes with σ^70^-dependent promoter DNA

**Figure 2:**
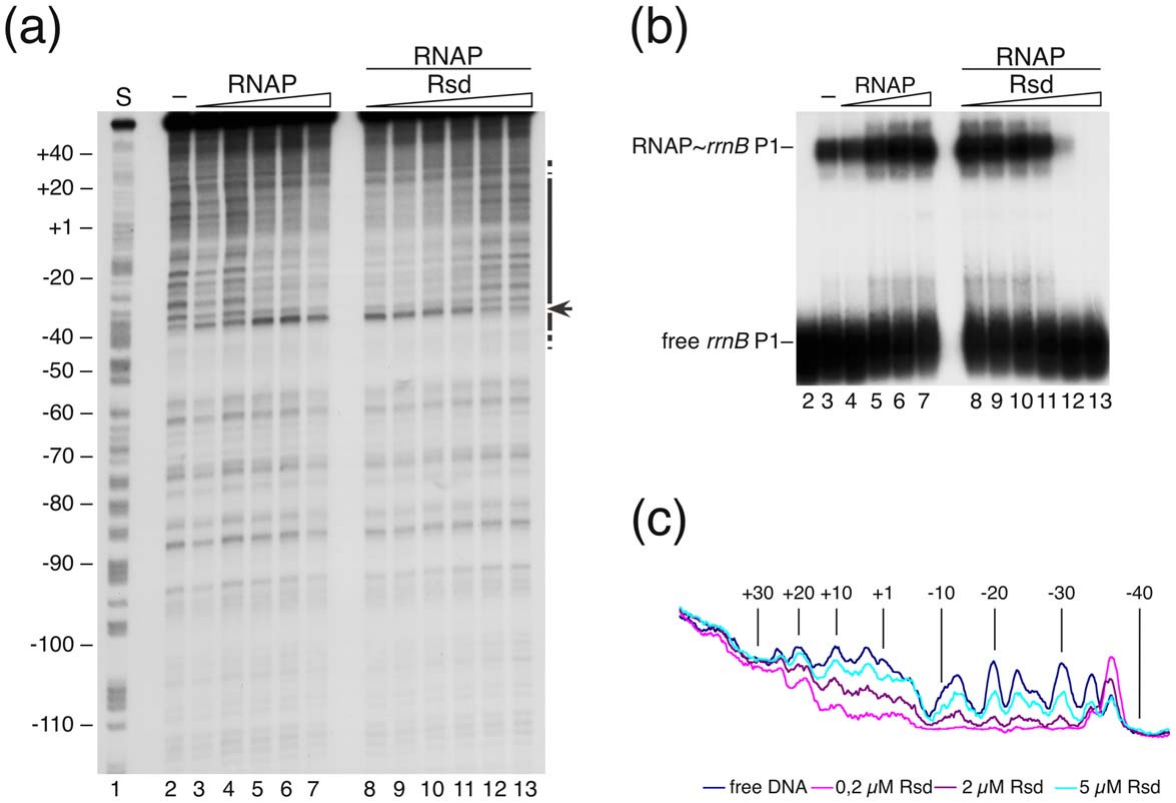
DNase I footprint analysis of RNA polymerase∼*rrnB* P1 promoter complexes: effect of increasing Rsd concentrations. (a) A denaturing footprint gel of the coding strand is shown. In the left part of the gel (lanes 2 to 7) open promoter complexes formed with increasing amounts of RNA polymerase were analyzed in the absence of Rsd. RNA polymerase concentrations were as follows: lane 2, none; in lanes 3 to 7 increasing concentrations of 10, 20, 50, 100, 200 nM RNA polymerase were employed, respectively. In lanes 8 to 13 promoter complexes were formed with 100 nM RNA polymerase each and increasing concentrations of 0.2, 0.5, 1, 2, 5 and 10 µM Rsd, respectively before DNase I treatment. In lane 1 an A+G sequencing reaction of the promoter fragment was separated. The region of DNase protection is marked by a vertical bar. A hypersensitive position is indicated by an arrow. (b) Aliquots of the complexes were separated on a native gel prior to DNase I treatment to verify the actual amount of complexes formed. Gel lanes refer to the same lanes shown in (a). (c) Densitometric profile of the band intensities from the gel shown in (a). The different lanes 2, 8, 11 and 12, respectively are indicated by the colours given in the key below.

We analyzed the effect of Rsd on transcription initiation complex formation of Eσ^70^ and Eσ^38^ with their corresponding promoters by gel retardation and footprinting experiments. Initial gel retardation studies with labeled promoter fragments revealed that increasing concentrations of Rsd inhibited binding of both holoenzymes to the appropriate promoters in a similar way (data not shown). To investigate potential structural rearrangements of the RNA polymerase promoter complexes in presence of Rsd we performed DNase I and KMnO_4_ footprint experiments. A typical DNase I footprinting result for Eσ^70^ RNA polymerase and the *rrnB* P1 promoter is shown in Figure 2. Prior to the treatment with DNase I, aliquots of the samples shown in Figure 2a were separated on a native polyacrylamide gel to ensure stable complex formation (Figure 2b). In the left panel (Figure 2a, lanes 2 to 7) DNase I accessibility to the *rrnB* P1 promoter DNA was analyzed at increasing concentrations of Eσ^70^ RNA polymerase holoenzyme. Polymerase binding protects DNA from cleavage resulting in a well-established footprint pattern consistent with previously published *rrnB* P1 footprints [34]. With increasing concentrations of RNA polymerase the protection pattern of the footprint, extending from +20 to −50 relative to the +1 transcription start of the *rrnB* P1 promoter, and a hyperreactive site at −37 becomes intensified (lanes 2 to 7). In the right panel the RNA polymerase concentration was set 100 nM and the samples were supplemented with increasing concentrations of Rsd. The addition of higher amounts of Rsd reduces the intensity of the footprint pattern, resulting in almost a total disappearance of the footprint and also the retardation pattern at the highest Rsd concentrations (Figure 2a and b, lanes 8–13). The results indicate that Rsd interferes with the ability of Eσ^70^ RNA polymerase holoenzyme to form complexes with the *rrnB* P1 promoter, *in vitro*. For a more detailed view the band intensities of lanes 2, 8, 11 and 12 of Figure 2a are presented as densitometric scans in Figure 2c. The nucleotide positions correspond to the positions given in Figure 2a.

To unravel at what stage of transcription initiation Rsd-dependent inhibition occurs we conducted a KMnO_4_ modification analysis, which can distinguish between open and closed promoter complexes by virtue of the single strand-specificity of the KMnO_4_ modification reaction [35]. The results clearly show that Rsd can effectively inhibit the formation of open complexes at the σ^70^-dependent *rrnB* P1 promoter *in vitro* (Figure S1). In summary, the analysis is consistent with the view that Rsd inhibits the formation of a functional Eσ^70^ transcription complex rather than causing a structural rearrangement of the initiation complex.

#### Rsd can also disturb the interactions of the Eσ^38^ holoenzyme with an appropriate promoter *in vitro*

**Figure 3:**
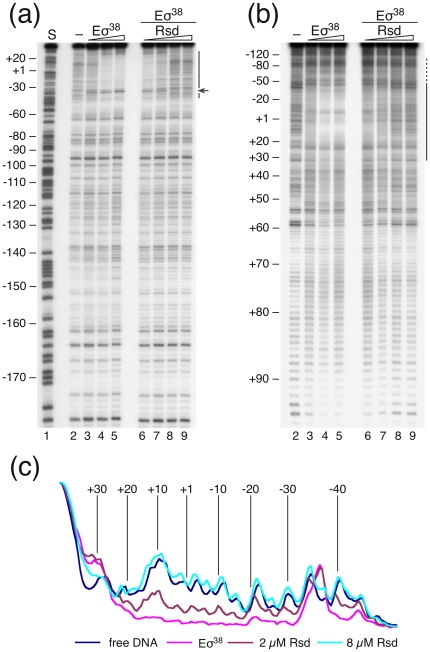
DNase I footprint analysis of Eσ^38^∼*bolA* P1 promoter complexes: effect of increasing Rsd concentrations. Footprints of the coding strand (a) and the non-coding strand (b) with different amounts of Eσ^38^ holoenzyme and Rsd are shown; lanes 2: no polymerase, lanes 3: 100 nM, lanes 4: 250 nM, lanes 5: 500 nM Eσ^38^, respectively. Lanes 6 to 9 contain 250 nM Eσ^38^ each, in presence of 1, 2, 4 and 8 µM of Rsd, respectively. Regions of protection are marked by vertical solid or broken lines for strong or weak protection, respectively. Numbers at the margin indicate nucleotide positions relative to the *bolA* P1 transcription start site. A hypersensitive position is marked by an arrow. Lane 1 contains an A+G sequencing reaction. (c) The densitometric profiles of the footprint lanes 2, 4, 7 and 9, presented in (a), are shown. A colour key indicating the corresponding lanes is given at the bottom.

Since Rsd is considered to facilitate the switch in the use of sigma subunits when cells enter from exponential to stationary phase, one would anticipate that the activity of the stationary phase-specific sigma factor σ^38^ should not be affected by Rsd. To test this preposition we repeated the binding and footprinting analyses with Eσ^38^ RNA polymerase holoenzyme and the σ^38^-dependent *bolA* promoter. Results are exemplified in Figure 3, which shows a DNase I footprinting experiment performed with the Eσ^38^ RNA polymerase bound to the *bolA* P1 promoter DNA in presence and absence of Rsd. In the absence of Rsd increasing amounts of the Eσ^38^ RNA polymerase holoenzyme resulted in a clear footprint on both DNA strands between nucleotide positions +30 and −60, relative to the *bolA* P1 transcription start, largely consistent with previous studies [36] (Figure 3a and b, lanes 2 to 5). Unexpectedly, however, the addition of increasing amounts of Rsd caused a strong reduction of the footprint, much the same as it was noted in case of the Eσ^70^ initiation complex at the σ^70^-specific promoter (Figure 3a and b, lanes 6 to 9). The densitometric profile shown in Figure 3c underlines the conclusion that Rsd also interferes with Eσ^38^ RNA polymerase-promoter complex formation. As in case of the Eσ^70^-dependent promoter Rsd does not cause a rearrangement of the Eσ^38^ initiation complex structure. The same conclusion was reached when open complex formation was analyzed by KMnO_4_ footprinting (Figure S2). In the presence of increasing amounts of Rsd signals characteristic for open complexes disappear, demonstrating once again that *in vitro* open complex formation is likewise inhibited at σ^38^-dependent and σ^70^-dependent promoters.

The finding contrasts with the presumed specificity of Rsd but is consistent with the above cross-linking and affinity binding experiments indicating Rsd binding to σ^70^- and σ^38^. Moreover, the result might be explained by earlier studies, which have demonstrated that Rsd is able to bind to RNA polymerase core [13].

Note that the DNA fragment also contains the *bolA* P2 promoter. This promoter is σ^70^-specific, however and does not form notable complexes with the Eσ^38^ RNA polymerase holoenzyme (a very weak footprint can be seen in Figure 3b indicated by a dotted vertical line). To verify the above result we repeated the binding competition experiment with a DNA fragment that contains the single *fic* promoter, which is strictly Eσ^38^-dependent. Complex formation between the *fic* promoter DNA and Eσ^38^ holoenzyme was challenged with increasing amounts of Rsd (see Supplementary Figure S3). In support of the above observation we noted a 50% reduction of the complex formed at 1 µM of Rsd and no complexes were remaining at the second highest Rsd concentration (4 µM).

#### Does Rsd differentiate between σ^70^- and σ^38^-dependent promoters under competitive *in vitro* transcription conditions?

**Figure 4:**
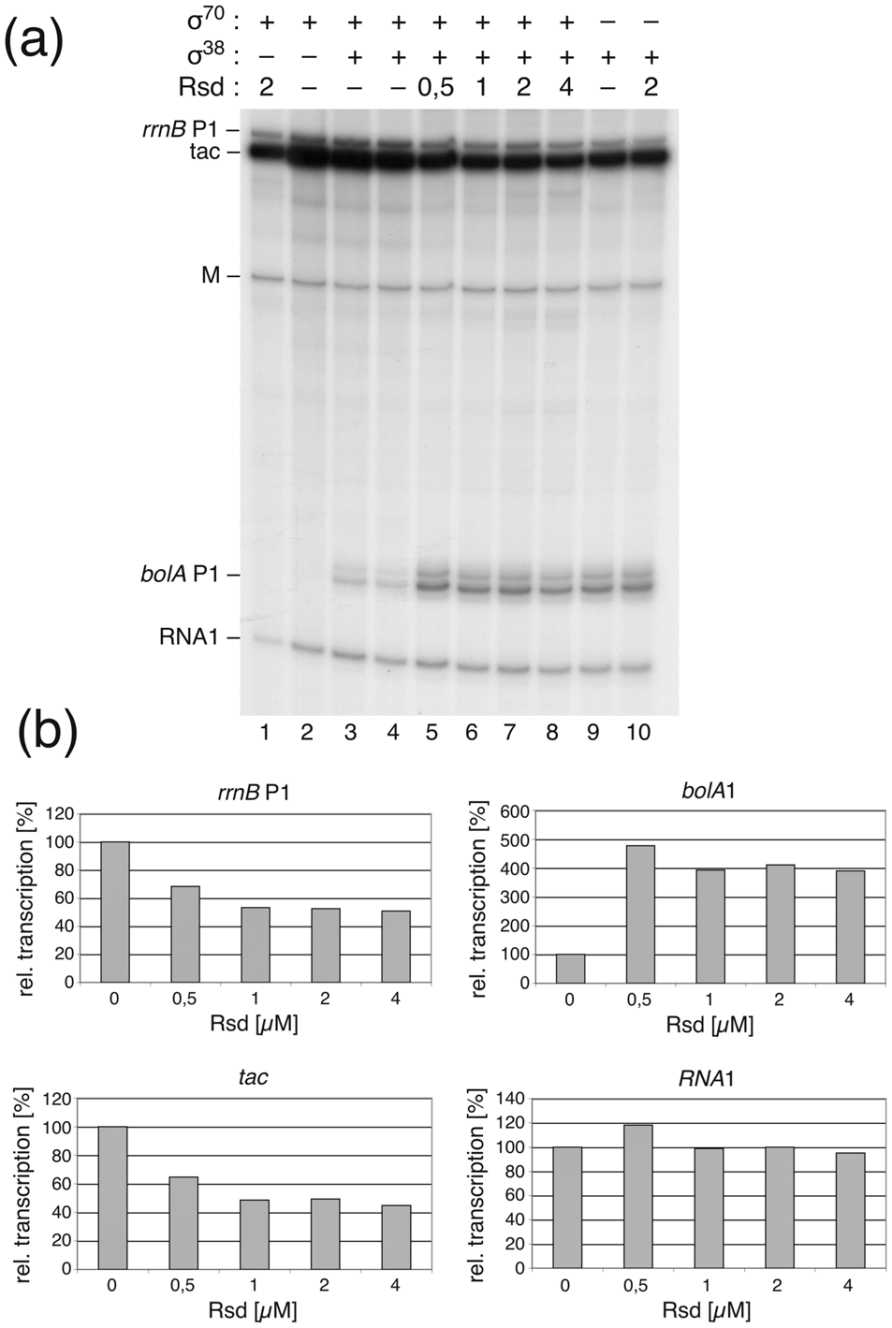
Effects of Rsd on *in vitro* transcription under competitive conditions. (a) Products from *in vitro* transcription reactions performed with RNA polymerase holoenzymes (20 nM) reconstituted with either 30 nM of σ^70^ or σ^38^ subunits (indicated by +) were separated on denaturing gels. Transcription products originating from the different promoters present on the template vector pSH666-1 (*rrnB* P1, tac, *bolA* P1, RNA 1) are indicated at the left margin of the autoradiogram. M denotes the position of a loading standard. The amount of Rsd (µM), when present in the reaction mixture, is given above the gel lanes. (b) Quantitative evaluation of the amounts of transcripts for promoters *rrnB* P1, *bolA*1, tac, and RNA 1 shown in (a, lanes 3 to 8). Bars represent relative transcripts as a function of the Rsd concentration present in the reaction mixture. Transcripts in the absence of Rsd (the mean from lanes 3 and 4) are set to 100%. The experiment was repeated twice with similar results.

The surprising observation from our *in vitro* binding and footprinting studies that Rsd, which is supposed to facilitate stationary phase-specific transcription, apparently affects both σ^70^- and σ^38^-dependent promoters in a similar way has provoked us to analyze the specificity of this regulator under conditions more closely reflecting the competitive situation in the cell. To this aim we performed *in vitro* transcription experiments with a multiple promoter template and the RNA polymerase holoenzymes Eσ^70^ and Eσ^38^ present alone or in combination. The system allows the simultaneous analysis of Rsd effects on several promoters with different specificity for single RNA polymerase holoenzymes and under conditions when Eσ^70^ and Eσ^38^ polymerases are competing each other. Since some promoters are known to be supercoil dependent the plasmid template pSH666-1 was used in its superhelical form to better match the *in vivo* conditions. The template vector used (pSH666-1) harbours the σ^70^-dependent promoters *rrnB* P1, tac, the RNA1 promoter as well as the weak *bolA* P2 promoter. The latter normally does not give rise to measurable transcripts under the conditions tested. In addition, the template vector contains the σ^38^-dependent *bolA* P1 promoter. All promoters give rise to transcripts of a defined length due to the tandem *rrnB* terminators T1T2, which have been cloned at defined distances downstream of the respective promoters. Transcription reactions directed only by the Eσ^70^ holoenzyme resulted in major products for the tac promoter followed by the *rrnB* P1 and the RNA1 promoters. When the Eσ^70^ holoenzyme was used as the only polymerase no transcripts could be detected for the *bolA* promoters (Figure 4a, lane 2). The addition of Rsd (2 µM) to the transcription mixture reduced the products for all promoters to about 50% (Figure 4a, compare lanes 1 and 2). Separate Rsd titration experiments with the Eσ^70^ holoenzyme revealed that 250 nM Rsd are already sufficient to reduce the amount of transcripts for the σ^70^-dependent promoters tac, *rrnB* P1 and the RNA1 promoters to a similar extent. It should be noted that transcription systems with single promoters yielded comparable inhibitions as observed in the binding studies (data not shown). Consistent with the expected specificity transcription with only the Eσ^38^ holoenzyme yielded a double band characteristic for the *bolA* P1 promoter (Reckendrees, unpublished). At the same time, the amounts of transcripts derived from the σ^70^-specific promoters are significantly lower, although the transcript from the tac promoter is still the strongest product under these conditions (Figure 4, lane 9). As opposed to the RNA polymerase binding studies to single promoters (Figure 2, 3 and Supplementary Figure S1, S2) the addition of 2 µM Rsd to the Eσ^38^ transcription system with multiple promoters did not change the transcript yields for any of the promoters significantly (Figure 4, lanes 9 and 10). A notable difference in the amount of the *bolA* P1 transcript is apparent, however, when both holoenzymes are present and Rsd is titrated to the reaction. Already at the lowest Rsd concentration (0.5 µM) the decreasing activity of the σ^70^-dependent promoters is compensated by a notable increase of the *bolA* P1 transcript (Figure 4, lanes 4 and 5). We conclude that under the conditions analyzed the *bolA* P1 promoter is sub-saturated by RNA polymerase. Under such conditions Rsd confers a competitive advantage on promoter selection by RNA polymerase. It is conceivable that a redistribution of the enzyme in favour of the σ^38^-dependent *bolA* P1 promoter occurs, following the inhibition of Eσ^70^-dependent promoter complex formation by Rsd. Since the RNA1 promoter is recognized by both Eσ^70^ and Eσ^38^ holoenzymes the simultaneous use of both polymerases does not show a competitive variation (see Figure 4b, lower right diagram). A quantitative evaluation of the different transcripts under competitive conditions (corresponding to lanes 3 to 8) is presented in Figure 4b. The results strongly suggest that the presence of Rsd facilitates binding and transcription from the sub-saturated *bolA* P1 promoter under competitive RNA polymerase conditions about five-fold.

### Studies on Rsd Expression

The Rsd protein is considered to be metabolically stable without noticeable turnover. Moreover, the cellular concentrations of Rsd increase between exponential and stationary phase from roughly 3000 to 6000 copies per cell [14], yet details of the mechanisms that cause the observed accumulation are not known. A complete understanding of the function of Rsd, its interaction with RNA polymerase and its involvement in transcriptional adaptation to stationary phase actually requires detailed knowledge of the expression and regulation of the *rsd* gene itself. Because Rsd is considered to play a major role during the transition from exponential growth to stationary phase, we were interested to analyze the influence of other well-known growth phase regulators on the expression of the *rsd* gene.

**Figure 5:**
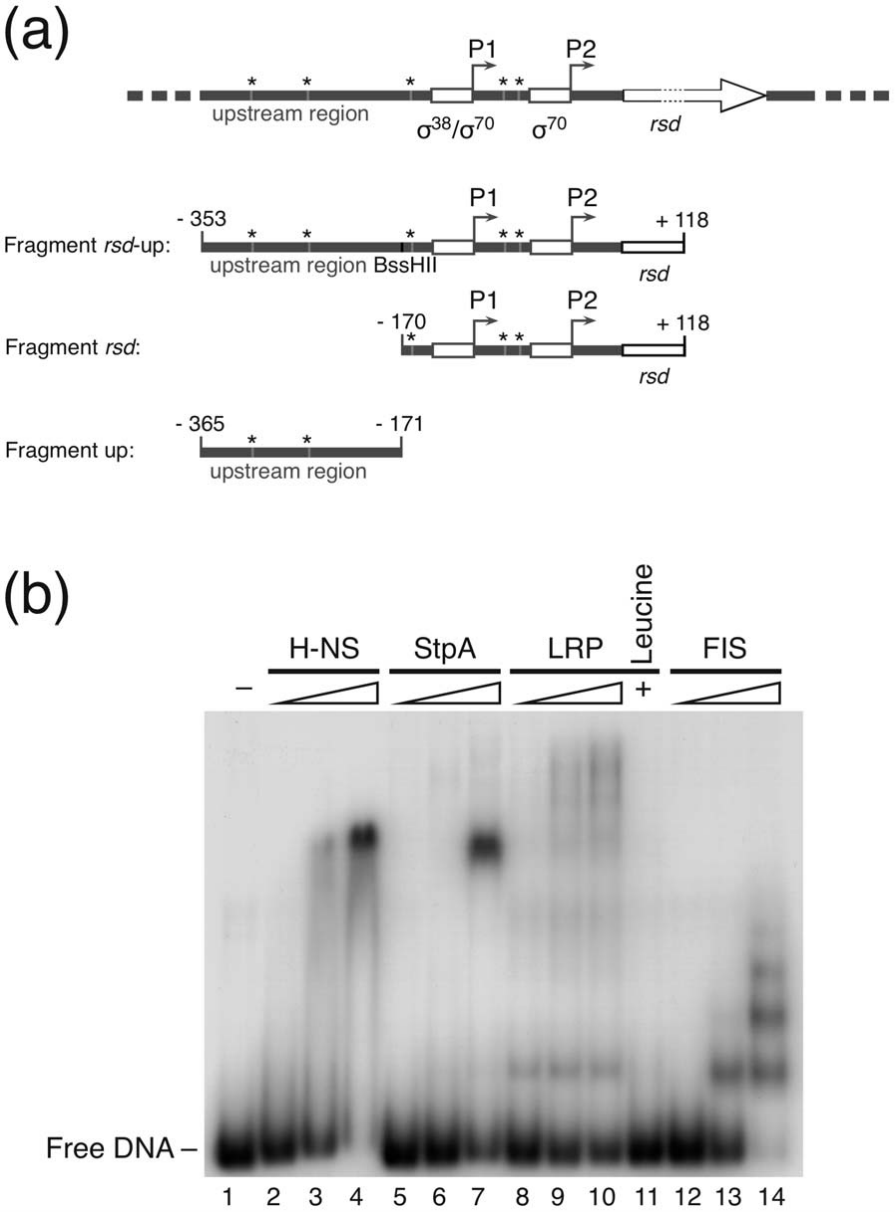
Binding of transcription factors to the *rsd*-promoter region. (a) The first line depicts a schematic representation of the *rsd* gene (open arrow) arrangement with the two promoters P1 and P2 (open boxes) with their upstream regulatory region. The figure is not drawn to scale. Arrows indicate transcription start points and the direction of transcription. The sigma factor specificities for the respective promoters are marked by σ^70^ or σ^38^, respectively. Asterisks indicate the positions of GATC *dam* methylation sequences. The lines below indicate different fragments (*rsd*-up, *rsd* and the up fragment) isolated for binding and footprintig studies. The sequence positions of the fragment ends relative to the transcription start site of the *rsd* P2 promoter are indicated. (b) Autoradiogram of a retardation analysis with the *rsd* fragment. Increasing concentrations of the NAP transcription factors were employed for binding: lane 1: no protein; lanes 2 to 4: H-NS (2 µM, 4 µM and 6 µM, respectively); lanes 5 to 7: StpA (0.5 µM, 2 µM and 6 µM, respectively); lanes 8 to 10: LRP (0.25 µM, 1 µM and 2 µM, respectively); lane 11: LRP (2 µM together with 30 mM leucine); lanes 12 to 14: FIS (0.1 µM, 0.5 µM and 2 µM, respectively).

Transcription of the *rsd* gene is controlled by two promoters, the distal σ^38^-dependent P1 and the more downstream σ^70^-dependent gearbox-type P2 promoter [11]. We have isolated the corresponding DNA fragment carrying both promoters together with a putative 210 bp upstream regulatory region to study potential binding of *E. coli* transcription factors known to regulate growth phase adaptation. A schematic arrangement of the two promoters including the upstream regulatory region and a scheme of the promoter fragments used for analyses is depicted in Figure 5a.

#### Binding of regulatory proteins to the *rsd* promoters

A set of small nucleoid associated proteins, termed NAPs, has recently been shown to be of major importance for the expression of many growth phase-related genes [37]. It had been demonstrated recently that a subset of these proteins is involved in the expression of the stationary phase-specific regulator 6S RNA [10]. In a first attempt we therefore analyzed if some of the most prominent NAPs, such as H-NS, StpA, LRP and FIS were able to bind to the *rsd* promoter region. We could show by gel retardation that all four proteins bind in a concentration-dependent way to a DNA fragment (*rsd* fragment, Figure 5a) harbouring the sequence of both *rsd* promoters P1 and P2 without the upstream region (Figure 5b). The two related proteins H-NS and StpA formed large complexes (Figure 5b, lane 3 and 7) while the interaction of LRP and FIS resulted in multiple bands with concentration-dependent successively increasing occupancy (Figure 5b, lane 8 to 14). Because it is known that the amino acid leucine modulates the specific binding of LRP on many regulatory DNA sites [21], [38] we also tested binding of LRP in the presence of 30 mM leucine. It turned out that the presence of the amino acid almost completely inhibited the binding of LRP to the promoter DNA (Figure 5b, lane 11).

#### Localization of transcription factor binding sites at the *rsd* promoter region

**Figure 6:**
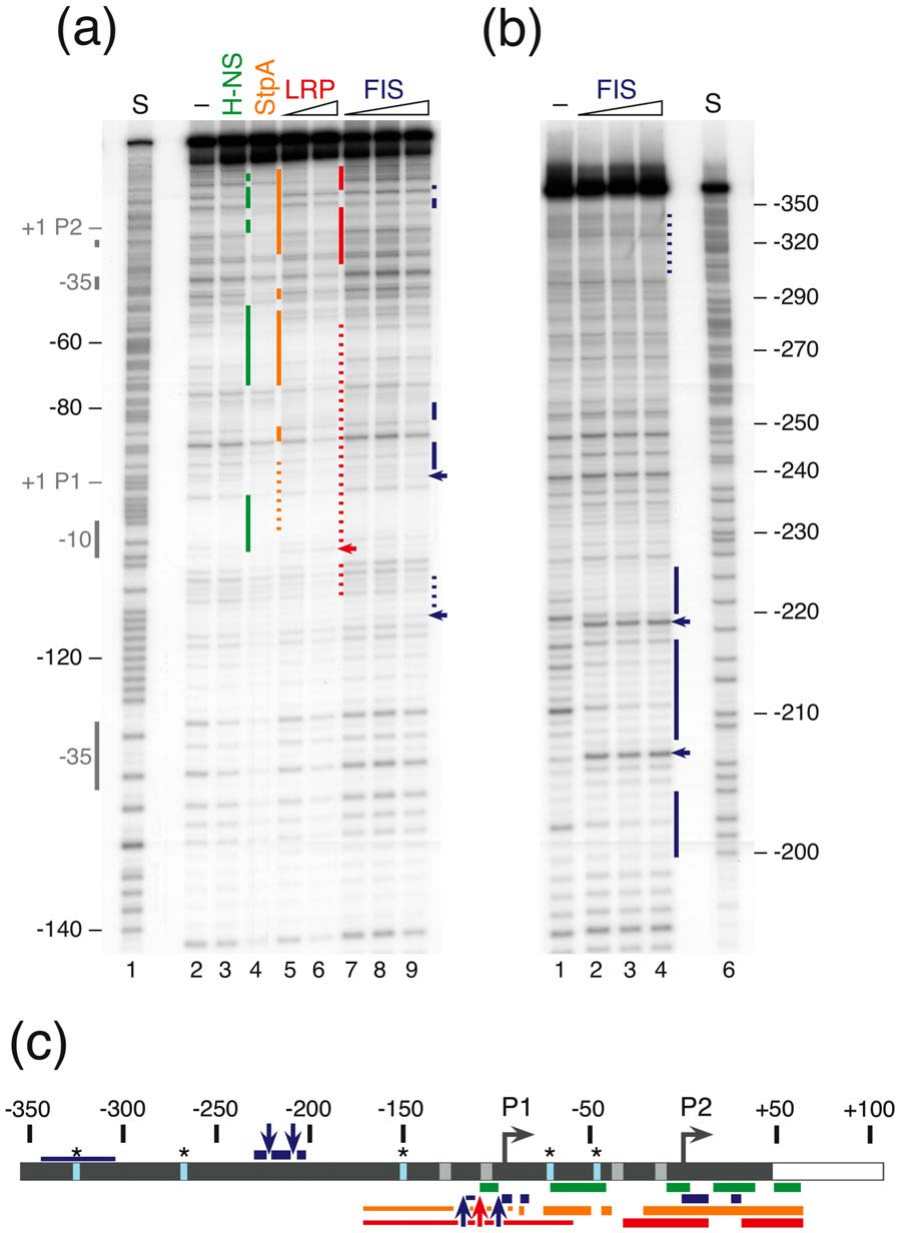
Localization of the binding sites for nucleoid associated proteins on the *rsd*-promoters. DNase I footprinting in presence of NAPs has been performed with the *rsd*-fragment (a) and the up-fragment (b). (a) The coding strand is presented. Lane 2 shows the separation of DNase I hydrolysis products in the absence of protein. The following proteins were used for binding: lane 3, 8 µM H-NS; lane 4, 8 µM StpA; lanes 5 to 6, 5 µM and 10 µM LRP; lane 7 to 9, 1 µM, 2 µM and 4 µM FIS, respectively. Regions of defined protection are indicated by coloured vertical lines next to the lanes (green: H-NS, orange: StpA, red: LRP and dark blue: FIS). Weak and extended (possibly non-specific) protections are indicated by broken lines. Hypersensitive DNase I sites are indicated by arrows. In lane 1 (S) an A+G sequencing reaction of the *rsd*-fragment is shown. Sequence positions and the P1 and P2 promoter core elements (−10, −35 and the transcription start sites +1) are denoted at the left margin. (b) A DNase I footprint analysis of the FIS binding sites on the up fragment is shown. The non-coding strand is presented. Lane 1 shows the separation of DNase I hydrolysis products in the absence of protein. In lanes 2 to 4 increasing concentrations of FIS (1 µM, 2 µM and 4 µM, respectively) were employed. In lane 6 (S) an A+G sequencing reaction of the up-fragment is shown. Regions of defined FIS-dependent protection are indicated by blue vertical lines, weak protection by broken lines. Hypersensitive positions are marked by arrows. (c) A schematic summary of the NAP binding sites within the *rsd* P1, P2 promoter upstream region according to the footprint data presented in (a) and (b) is shown. Regions of delimited protection are indicated by thick horizontal lines. Regions of weak and extended protection are indicated by thin lines. Hypersensitive positions are marked by arrows. The colour code is the same as in (a) and (b). Vertical light blue lines and an asterisk mark GATC *dam* methylation sites. The P1 and P2 start sites are marked by arrows and the promoter core elements (−10 and –35 regions) are indicated as light grey boxes. Sequence positions relative to the *rsd* P2 promoter start site are given on top.

To explore the exact binding regions of the NAPs we performed DNase I footprint experiments with the individual DNA-protein complexes. Binding was analyzed for the coding strand of the *rsd*-fragment (Figure 5a), except for FIS, where the analysis was also performed for the non-coding strand of the up-fragment (Figure 5a). Binding was performed in presence of heparin as competitor and suitable protein concentrations were determined in pilot experiments. A representative example of the footprint analyses is shown in Figure 6 and the data are summarized in Figure S4.

For H-NS several distinct sites in the region −107 to +63, relative to the transcription start of the *rsd* P2 promoter, can be identified, which are protected from DNase I cleavage (Figure 6a, lane 3). The binding sites overlap and flank the promoter core sequences of P1 and P2, respectively. One binding site is located in the −10 region of the *rsd* P1 promoter, a strong site of protection is downstream of the P1 start and upstream of P2. This site closely fits a predicted H-NS consensus sequence [39] (Figure S4). Two additional H-NS binding sites are located at the −10 region and the start site of the *rsd* P2 promoter.

For the two proteins StpA and LRP it is difficult to define distinct binding sites because both proteins have the tendency to cause extended protections from DNase I cleavage. Binding of the two proteins therefore results in almost a complete coverage of the DNA with a slight preference for the P1/P2 promoter core regions. Moreover, in the presence of LRP an enhanced cleavage occurs at a site overlapping with the −10 region of the P1 promoter. This site, together with a position upstream of −50 fit the predicted LRP consensus and may therefore act as potential nucleation sites [40].

Binding of FIS to the *rsd* fragment results in the characteristic pattern of protections flanked by hyperreactive cleavage sites [10]. Two binding sites are present, flanking each the P2 and the P1 promoters, overlapping the transcription start sites. On the non-coding strand additional FIS-dependent protections and two hypersensitive sites can be identified between positions −200 to −225, relative to the transcription start site of the P2 promoter (Figure 6b). The latter and the site close to P2 match predicted FIS consensus sites with a high score [40] (Figure S4). Whether these sites are relevant for *rsd* expression is not clear since they are intergenic within the divergently transcribed *nudC* gene, encoding NADH pyrophosphatase. A summary of the footprint results is presented in Figure 6c.

#### Does *dam* methylation affect the activity of the *rsd* promoters?

Inspection of the DNA sequence flanking the two *rsd* promoters revealed a striking frequency of GATC sites known as recognition sequences for the methylation by deoxyadenosine methyltransferase (Dam). There are 5 GATC sequences within a short DNA stretch (350 bp) upstream and between the *rsd* P1 and P2 promoters (Figure 5a). Dam methylation, next to mismatch repair, is known to be important for NAP-dependent epigenetic regulation, e.g. the LRP-dependent mechanism of phase variation during pili expression [41]. Moreover, it is known that Dam methylation contributes particularly to the regulation of genes linked to the stress response, such as the SOS response, or genes involved in amino acid and nucleotide metabolism or important for aerobic and anaerobic respiration, flagellar synthesis and chemotaxis [42]. Since the anti-sigma factor Rsd also belongs to the stress response family, we envisioned that the accumulation of GATC target sites for Dam-dependent methylation flanking the *rsd* promoters might indicate a link to a hitherto unknown layer of regulation. To check this hypothesis we first analyzed if the binding of the regulatory proteins H-NS, StpA, LRP and FIS to the *rsd* promoters was affected by DNA methylation. To this aim we isolated DNA fragments from cells with a defect in the *dam* gene (JM110). The presence or absence of the methylation at GATC sites was verified by restriction analysis with MboI. NAP binding was then compared to the methylated and non-methylated DNA fragments. No notable qualitative or quantitative differences in binding of the four proteins H-NS, StpA, LRP and FIS could be detected, however, suggesting that the methyl groups do not affect the specific binding of the NAPs to the *rsd* promoter region (data not shown).

**Figure 7:**
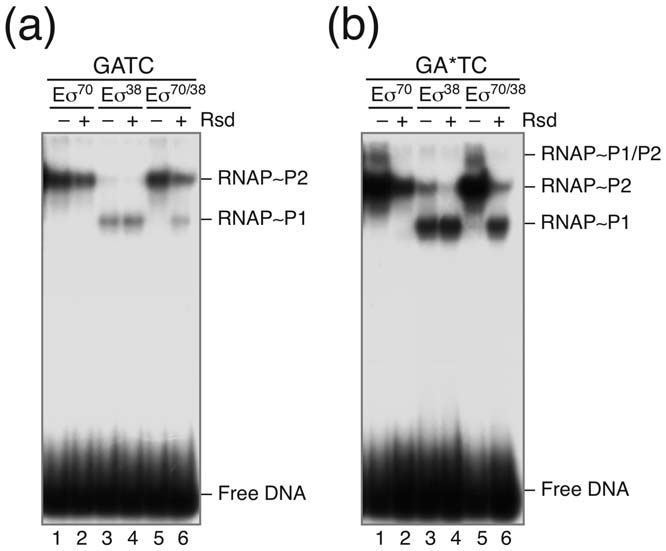
Effect of DNA-methylation on RNA polymerase binding to the *rsd* promoters P1 and P2. In (a) the non-methylated, indicated by GATC, and in (b) the methylated, indicated by GA*TC, *rsd*-fragments were used for complex formation with 100 nM each of the single RNA polymerase holoenzyms. The single holoenzymes Eσ^70^ (lanes 1–2) and Eσ^38^ (lanes 3–4), or a combination of both enzymes (lanes 5–6) were employed as indicated above the gel lanes. Complex formation of the different reactions was challenged by the addition of 2 µM Rsd (lanes 2, 4 and 6). The positions of the free *rsd*-fragment and the RNA polymerase P1, P2 or the double occupied P1/P2 complexes are indicated at the margin.

Next we asked if binding of the different RNA polymerase holoenzymes Eσ^70^ and Eσ^38^ might be affected by Dam-specific methylation. Again, we used non-methylated and methylated DNA templates containing both *rsd* promoters P1 and P2 for gel retardation. Binding was performed with the two holoenzymes Eσ^70^ and Eσ^38^, separately and in combination. Moreover, binding of the different holoenzymes was analyzed in the presence and absence of Rsd. Results are shown in Figure 7. With the non-methylated DNA the Eσ^70^ holoenzyme binds exclusively to the σ^70^-dependent *rsd* P2 promoter (Figure 7a, lane 1), whereas the Eσ^38^ holoenzyme binds preferentially to the σ^38^-dependent *rsd* P1 promoter and only weakly to the *rsd* P2 promoter (Figure 7a, lane 3). The addition of Rsd reduces the amount of the Eσ^70^∼*rsd* P2 complex significantly but has no effect on the intensity of the Eσ^38^∼*rsd* P1 complex band (Figure 7a, lanes 2 and 4). In presence of equimolar amounts of both sigma factors, the Eσ^70^ holoenzyme is formed preferentially, resulting almost exclusively in the formation of an Eσ^70^∼*rsd* P2 complex (Figure 7a, lane 5). When Rsd is present in this mixture a large fraction of the σ^70^ subunit is inactivated and binding of RNA polymerase to the P2 promoter is significantly inhibited. Concomitantly, a notable amount of Eσ^38^∼*rsd* P1 complex is now apparent (Figure 7a, lane 6). The results underline the specificity of Rsd, which, under competitive conditions, causes a selective preference of RNA polymerase for Eσ^38^-dependent promoters. This conclusion is consistent with the *in vitro* transcription assay shown before (Figure 4).

When the methylated DNA template was used binding of the different RNA polymerase holoenzymes was generally stronger (Figure 7b). The specificity of the different holoenzymes and the effect of Rsd was comparable, with the notable exception that addition of Rsd under competitive sigma factor conditions now resulted in much higher occupancy of the *rsd* P1 promoter, while the σ^70^-dependent P2 promoter becomes selectively inhibited (Figure 7b, lane 6; Table S2). The results are consistent with the conclusion that methylation at the GATC sites of the *rsd* promoters generally enhances RNA polymerase binding but specifically increases the preference of the Eσ^38^ holoenzyme to bind to the *rsd* P1 promoter. GATC-dependent methylation enhances the change in sigma factor specificity through a strong inhibition of the σ^70^-dependent P2 promoter in favour of the σ^38^-dependent P1 promoter. This effect is direct and not conferred by an affinity change of transcription factors (NAPs) but very likely by altering sigma factor competition at the *rsd* promoters through the activity of Rsd. In summary, the results demonstrate a contribution of DNA methylation on the regulation of *rsd* expression. By changing the RNA polymerase distribution between the σ^70^- and σ^38^-dependent *rsd* promoters Rsd is itself involved in the altered expression (autoregulation). We conclude that the DNA methylation state very likely has an effect on the Rsd responsiveness of certain promoters. The observation that transcription of *rsd* appears to increase, when DNA is methylated, might also suggest a link between the Rsd expression and the cell cycle.

### Analyses of Different Growth Phase Regulators on the *in vivo* Activity of the *rsd* Promoters

#### Effects of different growth rates

Since the *rsd* P2 promoter has the typical characteristics of gearbox promoters, whose activity often correlates inversely with the growth rate [43] we determined the relative *in vivo* promoter activities at different growth rates. To this aim we used a vector-based expression system (p*rsd*-up-cat), where the *rsd* promoters and their upstream regulatory region was fused to a promoter-less *cat* gene and transcript levels of transformed MG1655 cells were determined by primer extension analysis. The amount of the plasmid-encoded RNA1 transcript served as internal control. Total RNA was extracted at exponential and stationary phase from cells grown in different media with growth rates of µ = 0.35, µ = 0.95 and µ = 2.7 and subjected to primer extension analysis. The P2-derived products represented the predominant fraction of the *rsd* transcripts. The results obtained were fully consistent with the gearbox nature of the P2 promoter reported earlier [11]. Transcripts derived from the P1 promoter were negligible at the highest growth rate and reached only a fraction of the P2 transcript levels at the lower growth rates, indicating that their contribution to *rsd* expression is only marginal at the tested conditions.

To analyze the effects of a variety of cellular growth rate regulators we employed the same primer extension analysis described above and compared the amount of *rsd* promoter-derived transcripts from wild-type strains and strains with mutations in the genes for a number of selected regulators, which had been transformed with the *rsd* promoter vector p*rsd*-up-cat.

Strains with mutations in the *relA* and *dksA* genes were selected to analyse effects of the stringent control and growth rate regulator ppGpp. Both effectors are known to act as synergistic regulators [44]. A mutant in the *rsd* gene served to test for possible autoregulation. An *rpoS* mutant was selected to determine the effect of the stationary phase master regulator. Mutants in the NAP genes *hns*, *fis*, *lrp*, *stpA* were chosen to verify the *in vitro* binding results. A mutant with a defect in the *ssrS* gene encoding the regulatory 6S RNA was selected for its functional homology to *rsd*. Finally, we selected a strain with the *dam* mutation to verify the difference in RNA polymerase binding observed with methylated and non-methylated DNA *in vitro* (Figure 7).

Since the amounts of transcripts derived from the *rsd* P1 promoter were usually negligible, while the major activity was always found for the P2-derived transcripts, we concentrated our studies on the latter promoter. A brief summary of results for the *rsd* P1 promoter is presented in Figure S5. For strains with defects in the NAP-encoding genes *lrp* and *stpA* we did not find reproducible differences in the amount of *rsd* transcripts compared to their isogenic wild-type strains. This is in line with the rather non-specific binding that was observed in the footprint analysis (Figure 6). In addition, deletion of the gene for the riboregulator 6S RNA (*ssrS*) did not result in an altered *rsd* mRNA level. This is of special interest because 6S RNA itself is a regulator, considered to facilitate the switch in specificity between exponential and stationary growth transcription by interfering with the Eσ^70^ holoenzyme [45], [46]. Hence, 6S RNA can be regarded as a functional homolog of Rsd [47]. The finding that *rsd* transcription is not altered in the *ssrS* mutant contrasts with earlier studies performed under long-time stationary growth, where the *rsd* P2 promoter was found to be negatively affected by 6S RNA [48]. It is consistent, however with results from a microarray study performed with total RNA from early stationary growing cells [12] and a previous promoter analysis [49].

**Figure 8:**
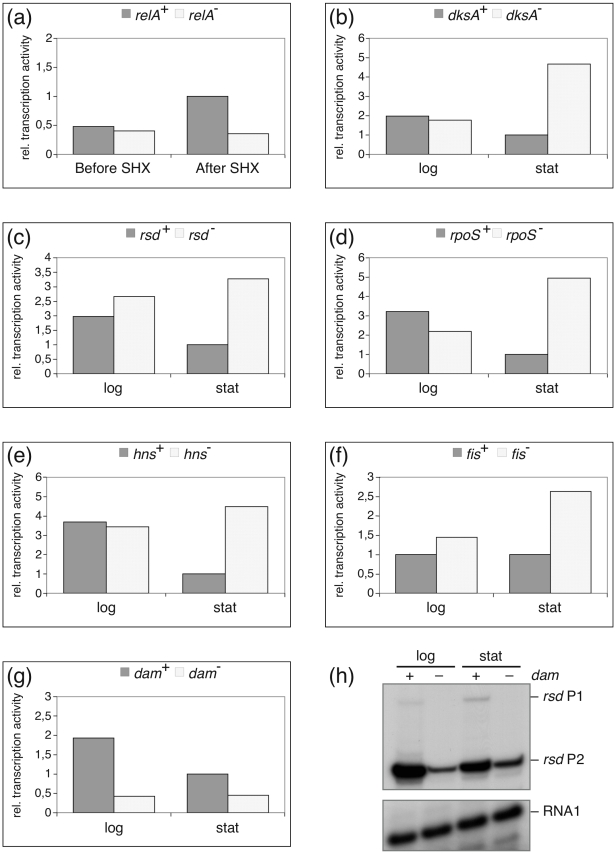
*In vivo* activity of the *rsd* P2 promoter in different strain background. Relative transcription activities of the *rsd* P2 promoter were determined by quantitative primer extension reaction from RNA extracted from strains with defects in defined growth rate regulatory genes. Total RNA was extracted during exponential (log) or stationary (stat) growth phases or before or after induction of the stringent response by serine hydroxamate (SHX). The diagrams represent relative amounts of P2-derived transcripts normalized to the RNA 1 transcript. The mean of two to three independent experiments is shown. In all comparisons the *rsd* P2 expression level during stationary phase was set to 1. RNAs from strains with mutations in the following genes were analyzed in comparison to the RNAs from their respective wild-types: (a) *relA*, (b) *dksA*, (c) *rsd*, (d) *rpoS*, (e) *hns*, (f) *fis*, (g) *dam*. For the different *E. coli* strains see Table S1. In (h) a representative example for the primer extension of the analysis from *dam* ^+^/*dam* ^−^ strains is shown. Primer extension products specific for RNA 1, *rsd* P2 and *rsd* P1 are indicated.

Results for the other regulators (*relA*, *dksA*, *rsd*, *rpoS*, *hns*, *fis* and *dam*) are summarized in Figure 8 a to g, where the relative P2-derived transcript levels are exemplified for different growth phases. An example for a typical primer extension result for the *dam* ^+/-^ strains is presented in Figure 8 h. The failure to synthesize high levels of ppGpp, following addition of serine hydroxamate, caused a strong reduction in P2 activity in the *relA*-deficient strain (Figure 8a). This result is consistent with the conclusion that the *rsd* P2 promoter is under positive stringent regulation reported previously [11]. Surprisingly, the lack of DksA, known as a synergistic co-regulator of the stringent response, had an opposite effect during the stationary phase (Figure 8b). It is possible that there is a compensatory enhancement of the basal ppGpp level in the *dksA* strain. Further experiments are required to resolve this interesting phenomenon. In an *rsd*-deficient strain we noticed a weak de-repression of the P2 activity during logarithmic growth and a significant activation at the stationary phase, consistent with the assumption that Rsd has an autoregulatory function in the cell (Figure 8c). The prominent effect on *rsd* P2 activity in absence of RpoS (σ^38^), the master regulator for stationary phase expression, is a dramatic increase in P2 activity during the stationary phase (Figure 8d). This reflects very likely an increased level of the Eσ^70^ holoenzyme under those conditions. During logarithmic growth the absence of RpoS caused only a slight reduction in P2 activity. Consistent with the results from the NAP binding and footprint studies (Figure 5 and 6) the absence of the two proteins H-NS and FIS resulted each in a clear de-repression during the stationary phase (Figure 8e and f). Finally, in the *dam* ^−^ strain we noticed a significant reduction of the *rsd* P2 activity at both growth phases (Figure 8g and h). This nicely supports the observations from the RNA polymerase binding studies to methylated or non-methylated *rsd* promoter DNA (Figure 7).

## Discussion

In the above study we show that Rsd is able to interact with RNA polymerase core enzyme and can form dimers. This is consistent with earlier reports [13], [14]. Dimers have also been shown to be the preferred molecular species of the related anti-sigma factor AsiA [27]. A recently discovered amino acid substitution (L18P) in Rsd destabilizes the dimer formation [29]. This mutation supports σ^38^-dependent transcription, which is consistent with the assumption that only the monomer binds to σ^70^. Hence, dimers may have a functional role in regulating the level of active Rsd molecules. Whether or not the extent of Rsd dimers is regulated in the cell by unknown effectors cannot be answered yet and requires further studies. The three-dimensional structure of Rsd in complex with σ^70^ region 4 reveals that the sigma-binding surface is connected to exposed cavities, which might act as binding pockets for small regulatory molecules. Our preliminary tests to find such putative effectors, (amino acids, cAMP etc.) were unsuccessful so far (data not shown).

Unexpectedly, we found evidence that the anti-sigma factor Rsd does not only interact with σ^70^ but can also associate with the stationary phase-specific sigma factor σ^38^ *in vitro*. This was demonstrated by cross-linking and independently by affinity binding. Whether this interaction is of functional importance was further analyzed by transcription studies, which showed that Rsd was able to inhibit open complex formation of both holoenzymes, Eσ^70^ and Eσ^38^, at their appropriate promoters. It should be noted that this was the case when the analyses were performed *in vitro* under non-competitive conditions. When the effect of Rsd was studied under more complex conditions, allowing competition between different sigma factors and promoter selection by RNA polymerase, it turned out that the presence of Rsd now changed transcription specificity in favour of Eσ^38^ holoenzyme and σ^38^- dependent promoters. The results indicate that the mode of action of Rsd is not governed by a strong affinity preference for the principal sigma factor σ^70^ but apparently involves a balanced change in concentration and affinity of additional interacting partners, involving the accessibility of competing promoters and σ factor competition. This is particularly reflected in the different results Rsd provokes in open complex formation experiments with single promoters and single RNA polymerases, presented in Figures 2 and 3, versus results obtained under competitive sigma factor conditions employing multiple promoter vectors, exemplified in Figure 4.

Generally, in a simplistic view, anti-sigma factors bind to sigma and preclude the formation of transcription-competent RNA polymerase holoenzymes. A notable exception is AsiA, which acts both as anti-sigma factor and, together with the regulator MotA, as co-activator [50]. Given the similarity in the interaction surfaces of σ^70^ for AsiA and Rsd one could speculate that Rsd may also exert functions other than sequestering the primary sigma factor σ^70^. In line with this our binding and footprinting studies are not fully consistent with the simple conception that Rsd fulfils its anti-sigma factor function only by reducing the active fraction of the respective sigma factor through binding. The mechanism how Rsd contributes to the shift in sigma factor usage and promoter selection is obviously more complex. It likely involves transient interactions of Rsd with RNA polymerase core, changes in the concentration of available reaction partners, including σ^70^ and σ^38^, but also putative Rsd dimer formation. Hence, the outcome of Rsd-dependent regulation is determined by an orchestrated interplay of numerous components involved in the transcription reaction.

In much the same way as the selectivity of Rsd for σ^70^ is not absolute the molecular reasons for the specificity difference between σ^70^ and σ^38^ to bind to RNA polymerase core and to select their cognate promoters cannot easily be discerned because both sigma factors share high structural homology. Numerous studies to understand the specificity difference of σ^70^ and σ^38^ have revealed only some clues to explain the divergent functions of these rather homologous sigma factors [51], [52], [53], [54], [55], [56], [57]. There are only a limited number of amino acid residues in the functional regions 2 and 4 of the two factors, which is not conserved. Among the putative promoter-discriminating elements between the two proteins are a few non-conserved amino acid residues in domains 4.2 and 2.4 and a small number of different amino acids located in subregions 2.1 and 3.2, which are important elements for RNA polymerase core interaction [55]. Region 4.2 is known to interact with bases of the −35 promoter element. Although the homology within that region is rather high many σ^38^-dependent promoters do not share good −35 sequence conservation, consistent with the minor role of this recognition element for stationary phase-specific promoters. Generally, σ^38^ and σ^70^-specific promoter sequences exhibit only limited structural differences. A conserved cytidin at position −13 is considered as presumably the most pronounced difference and appears to be a hallmark for σ^38^-dependent promoters [58]. Because region 2.4 makes contacts to nucleotides within the –10 promoter site and flanking nucleotides of the non-template strand this points to non-conserved amino acid residues within region 2.4 as discriminating elements between σ^38^ and σ^70^.

One might speculate that the same differences in the recognition domains of the two sigma factors may also account for the discrimination of Rsd to engage specifically with one of the two specificity factors. From the high resolution crystal structure of Rsd in complex with a C-terminal fragment of σ^70^ it was concluded that the binding of Rsd sterically not only interferes with the interaction of σ^70^ and the −35 promoter recognition element but also with the β flap, indicating that both the recognition of core polymerase as well as certain promoter structures may be influenced by Rsd [59]. Major points of contact between Rsd and σ^70^ have been identified in subregion 4.2. However, within the major recognition helix of this domain there are only three amino acids (D570, E591, A594), which differ between σ^70^ and σ^38^ among the positions identified in direct interaction with Rsd [59]. From the structural analysis of Rsd in complex with sigma region 4 it is also known that the interacting surface extends into subregion 4.1, yet the amino acid side chains in contact with Rsd are again conserved between σ^70^ and σ^38^. It has been shown that Rsd can interact simultaneously with σ^70^ regions 4 and 2, likely forming an extensive interface [60]. Moreover, additional amino acid residues in subregion 2.1, not present in σ^38^, and a low degree of amino acid conservation in subregion 3.2, are involved in core binding and may provide potential sides for a differential specificity to recognize Rsd. Unfortunately this region is not present in the crystal structure from which the contact sites have been derived. It is feasible, however, that binding of Rsd to these regions will interfere with RNA polymerase core binding. In summary, the high similarity between crucial domains of both sigma factors may explain the observations made in this study that under non-competitive conditions Rsd can recognize both, σ^70^ and σ^38^ (see Figures 2 and 3). Hence, the preferred specificity of Rsd to support σ^38^-dependent transcription may necessitate additional factors.

The two sigma factors, σ^70^ and σ^38^, certainly differ in their capacity to interact with activator proteins. Most of the activators known to affect σ^70^ are considered to bind to the subregion 4.2, in fact, in close proximity to the same amino acid residues that make contact to Rsd. Again, the amino acid sequence in σ^38^ is very similar, almost identical, within this domain and does not give an easy explanation how activator proteins may be differentiated. Obviously, the active target for activators is RNA polymerase holoenzyme, where the surface of sigma is altered by the core interaction.

A similarly complex mode of action has also been shown for other anti-sigma factors, such as AsiA, for example [61], [62], which is commonly assigned with Rsd and AlgQ to the same family of regulators [31]. Nevertheless, AsiA and Rsd, although they share similar binding properties to σ^70^ region 4.2, have a number of distinctly different activities [63]. Unlike AsiA, Rsd binding to region 4 does not alter the structural core of the helix-turn helix domain of σ^70^ [3], [62].

### Regulation of Rsd Expression

Down-regulation of the activity of σ^70^ by the anti-sigma factor Rsd at the onset of stationary growth immediately raises the question of how Rsd itself is regulated. Moreover, since Rsd targets an essential sigma factor it will be toxic for the cell if not regulated properly. Our detailed transcriptional analysis of the regulation of *rsd* expression is consistent with previous observations and underlines the gearbox characteristic of the *rsd* P2 promoter [11]. Moreover, binding and footprinting studies revealed that nucleoid-associated proteins, such as H-NS or FIS, known as growth phase effectors are involved in transcriptional control through specific binding to the *rsd* P1 and P2 upstream promoter region.

As a result of special importance we show here for the first time that methylation of a cluster of *dam* sites, present in the *rsd* regulatory region, enhances RNA polymerase binding to the *rsd* promoters. Methylation of these sites also changes the distribution between the holoenzymes Eσ^70^ and Eσ^38^ to initiate transcription from the *rsd* P1 or P2 promoters. The results strongly indicate that expression of the *rsd* gene is linked to the DNA methylation status of the cell. Consistent with highly methylated DNA during stationary phase, when cell division is slowed down, the Eσ^38^-dependent *rsd* P1 promoter becomes selectively activated.

The analysis of the *in vivo* activities of the *rsd* promoters at different growth phases in strains with mutations in the genes encoding different growth phase regulators confirmed the repressing effect of the nucleoid-associated proteins FIS and H-NS observed *in vitro*. Moreover, the study revealed that the activity of the *rsd* promoters is linked to the stationary phase network governed by RpoS as well as to the stringent control network mediated by ppGpp. It remains unclear, whether the latter effect is direct or results from the redistribution of RNA polymerases released from negatively regulated stable RNA promoters [64]. Interestingly, the transcription factor DksA, which normally acts synergistically with ppGpp, showed a repressing effect on *rsd* expression, indicating the possibility of a differential regulation by DksA and ppGpp. Such a differential action of both regulators has been observed in some other cases before [65], [66], [67].

A number of unresolved questions remain. For instance, deletions of the *rsd* gene do not show an apparent phenotype and over-expression causes only a small number of both σ^70^- and σ^38^-dependent genes to be differentially expressed [29]. A lack of apparent phenotypes is also characteristic for deletions of the riboregulator 6S RNA, which is a functional homologue to Rsd. As in case of 6S RNA it is astonishing that the cellular concentrations of Rsd are already rather high during exponential growth. The question if we have to expect alternative functions, not yet discovered, or whether Rsd function might be controlled through dimer formation has not been finally answered yet [14].

## Supporting Information

Figure S1 **KMnO_4_ footprint analysis of RNA polymerase∼** ***rrnB*** **P1 promoter complexes: effect of increasing Rsd concentrations.** Complexes of Eσ^70^ holoenzyme with the *rrnB* P1 promoter fragment were cleaved after KMnO_4_ treatment. The analysis of the coding strand is shown. In lanes 2 to 6 increasing polymerase concentrations were applied: lane 2, no polymerase, lanes 3 to 6: 20, 40, 100 and 200 nM RNA polymerase, respectively. Lane 1 shows the A+G sequence. Samples on lanes 7 to 12 contained 100 nM RNA polymerase each and 0, 0.5, 1, 2, 4 and 8 µM Rsd, respectively. Addition of RNA polymerase to *rrnB* P1 promoter DNA (lanes 2 to 6) resulted in the expected characteristic KMnO_4_ modification signal at positions -10T and -11T (marked on the right) indicative for a transcriptionally active open promoter complex [Supplementary Reference S1 in Text S1]. Nucleotide positions relative to the transcription start site of the *rrnB* P1 promoter are given at the left margin. An A+G sequencing reaction of the promoter DNA was separated in lane 1 (S).
(TIFF)

Figure S2 **KMnO_4_ footprint analysis of Eσ^38^∼** ***bolA*** **1 promoter complexes: effect of increasing Rsd concentrations.** Complexes of Eσ^38^ holoenzyme with the *bolA* P1 promoter fragment were cleaved after KMnO_4_ treatment. The analysis of the coding strand is shown in (a). In lanes 1 to 6 increasing polymerase concentrations were applied: lane 1, no polymerase, lanes 2 to 6: 5, 10, 20, 50 nM and 100 nM Eσ^38^ holoenzyme, respectively. Samples in lanes 7 to 12 contained 50 nM Eσ^38^ each and 0, 0.5, 1, 2, 4 and 8 µM Rsd, respectively. In Lane 13 an A+G sequencing reaction of the promoter fragment was separated. With increasing RNA polymerase concentration KMnO_4_-sensitive positions, indicating the presence of open complexes (-11T, -12A, -13G, are marked at the left margin), became visible. Nucleotide positions relative to the transcription start site of the *bolA* P1 promoter are given at the right margin. (b) Gel shift analysis of aliquots from the samples used for the KMnO4 footprint reaction shown in (a) prior to the modification. Lane numbers correspond to those shown in (a). The positions for the free DNA and the Eσ^38^∼*bolA*1 promoter complex are given on the left margin.
(TIFF)

Figure S3 **Effect of Rsd on Eσ^38^∼** ***fic*** **promoter complex formation.** (a) Binding of the RNA polymerase Eσ^38^ holoenzyme (200 nM) to a DNA fragment harbouring the σ^38^-dependent *fic* promoter was analyzed by gel retardation. Complex formation was challenged by increasing concentrations of Rsd. In lane 1 the free DNA is shown. Lane 2 represents the complex in the absence of Rsd. In lane 3 to 6 increasing Rsd concentrations of 1 µM (lane 3), 2 µM (lane 4), 4 µM (lane 5) and 8 µM (lane 6) were present. (b) Diagram showing the quantitative evaluation of the data from (a) indicating the remaining amounts of complex as a function of the Rsd concentration.
(TIFF)

Figure S4 **Sequence of the** ***rsd*** **promoter region.** The sequence given corresponds to the *rsd*-up fragment presented in Figure 5a. Numbers indicate sequence positions relative to the *rsd* P2 transcription start site. Promoter core elements (−10 and −35 regions) are boxed and highlighted with bold letters. Transcription start sites of the *rsd* P1 and P2 promoters are marked by bold-type capital letters. The Rsd translation initiation codon is shown in bold-type and boxed. The five GATC sites are bold-type and underlined. NAP binding sites and the respective colour code are taken from Figure 6. The thickness of the lines represents high or low affinity of the respective NAPs. Hyperreactive sites are indicated by arrows. Sequence positions matching the known consensus sites for H-NS, LRP and FIS [39], [40] are highlighted in bold-type with the colour given in the key of Figure 6. Overlapping sites between H-NS and LRP are shown in yellow colour.
(TIFF)

Figure S5 ***rsd*** **P1 promoter activity in different strain background during stationary phase of growth.** For the *rsd* P1 promoter notable amounts of transcript were only obtained during stationary phase of growth. The diagram depicts the relative amounts of *rsd* P1-derived transcripts normalized to the RNA 1. Compared are the transcripts from strains with defects in *relA*, *dksA*, *rsd*, *rpoS*, *hns*, *fis* and *dam* relative to the transcripts from the respective wild-type strains, normalized to 1. Shown is one representative experiment out of two to three with similar results.
(TIFF)

Table S1 **Bacterial strains and plasmids used in this study.**
(DOC)

Table S2 **Quantitative evaluation of RNA polymerase complexes formed with methylated and non-methylated** ***rsd*** **promoter DNA.**
(DOC)

Text S1 **Supplementary References.**
(DOC)
