# Supplementary figures and images for: The E. coli Anti-Sigma Factor Rsd: Studies on the Specificity and Regulation of Its Expression
Source: PLoS One. 2011 May 6;6(5):e19235. doi: 10.1371/journal.pone.0019235 (PMC3089606; doi:10.1371/journal.pone.0019235)

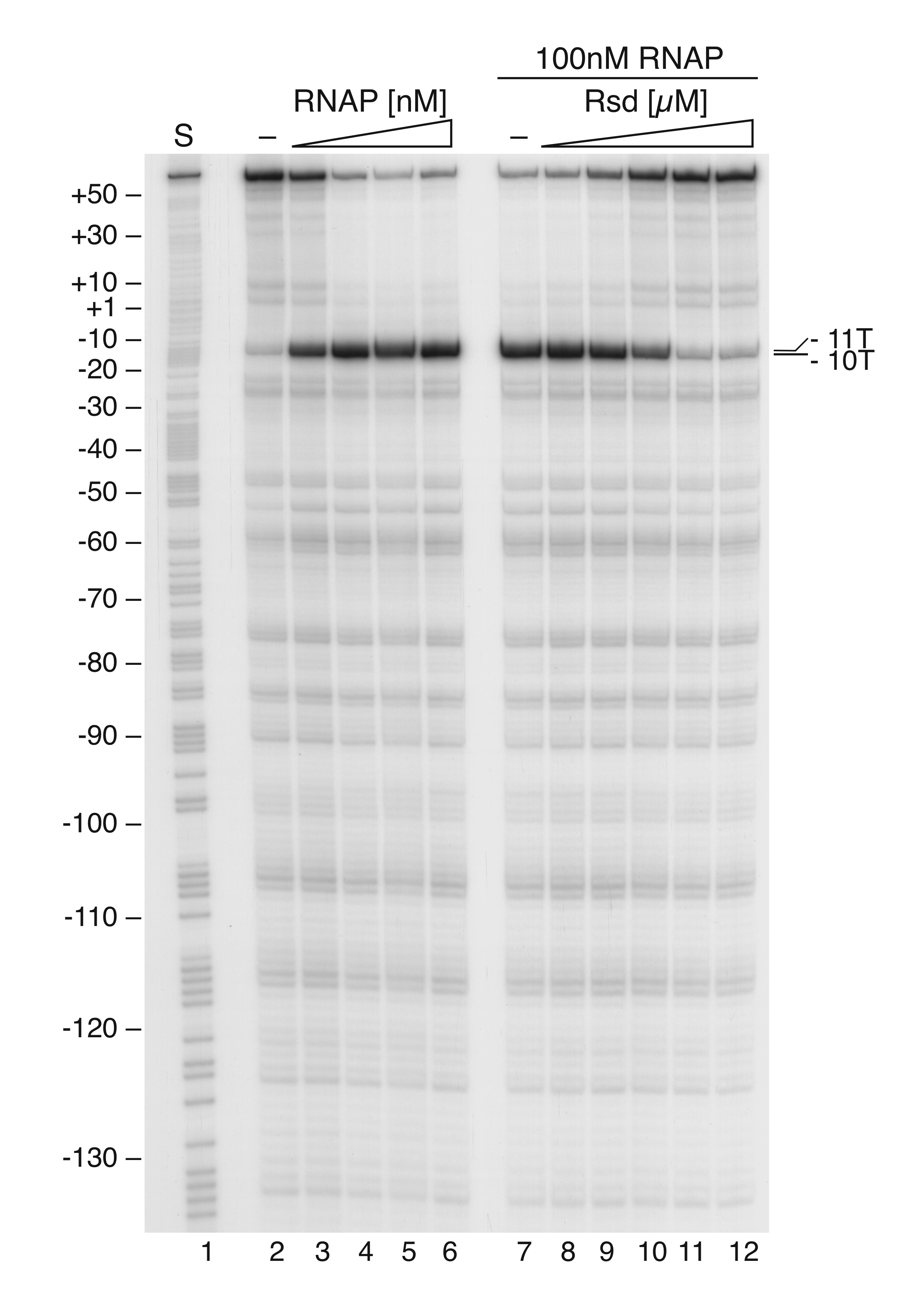

Supplement: Figure S1 — KMnO4 footprint analysis of RNA polymerase∼ rrnB P1 promoter complexes: effect of increasing Rsd concentrations. Complexes of Eσ70 holoenzyme with the rrnB P1 promoter fragment were cleaved after KMnO4 treatment. The analysis of the coding strand is shown. In lanes 2 to 6 increasing polymerase concentrations were applied: lane 2, no polymerase, lanes 3 to 6: 20, 40, 100 and 200 nM RNA polymerase, respectively. Lane 1 shows the A+G sequence. Samples on lanes 7 to 12 contained 100 nM RNA polymerase each and 0, 0.5, 1, 2, 4 and 8 µM Rsd, respectively. Addition of RNA polymerase to rrnB P1 promoter DNA (lanes 2 to 6) resulted in the expected characteristic KMnO4 modification signal at positions -10T and -11T (marked on the right) indicative for a transcriptionally active open promoter complex [Supplementary Reference S1 in Text S1]. Nucleotide positions relative to the transcription start site of the rrnB P1 promoter are given at the left margin. An A+G sequencing reaction of the promoter DNA was separated in lane 1 (S). (TIFF) [file pone.0019235.s001.tif]

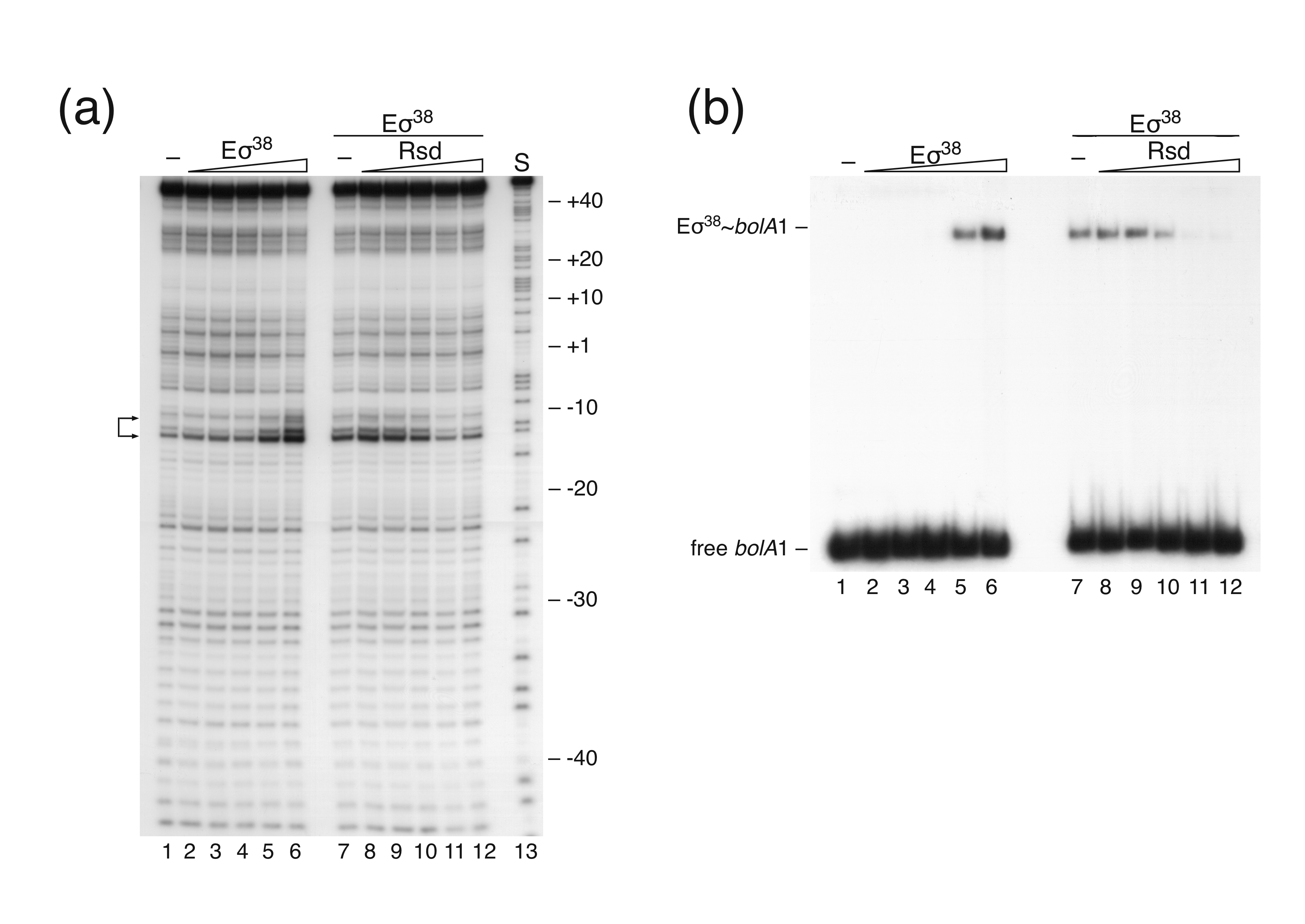

Supplement: Figure S2 — KMnO4 footprint analysis of Eσ38∼ bolA 1 promoter complexes: effect of increasing Rsd concentrations. Complexes of Eσ38 holoenzyme with the bolA P1 promoter fragment were cleaved after KMnO4 treatment. The analysis of the coding strand is shown in (a). In lanes 1 to 6 increasing polymerase concentrations were applied: lane 1, no polymerase, lanes 2 to 6: 5, 10, 20, 50 nM and 100 nM Eσ38 holoenzyme, respectively. Samples in lanes 7 to 12 contained 50 nM Eσ38 each and 0, 0.5, 1, 2, 4 and 8 µM Rsd, respectively. In Lane 13 an A+G sequencing reaction of the promoter fragment was separated. With increasing RNA polymerase concentration KMnO4-sensitive positions, indicating the presence of open complexes (-11T, -12A, -13G, are marked at the left margin), became visible. Nucleotide positions relative to the transcription start site of the bolA P1 promoter are given at the right margin. (b) Gel shift analysis of aliquots from the samples used for the KMnO4 footprint reaction shown in (a) prior to the modification. Lane numbers correspond to those shown in (a). The positions for the free DNA and the Eσ38∼bolA1 promoter complex are given on the left margin. (TIFF) [file pone.0019235.s002.tif]

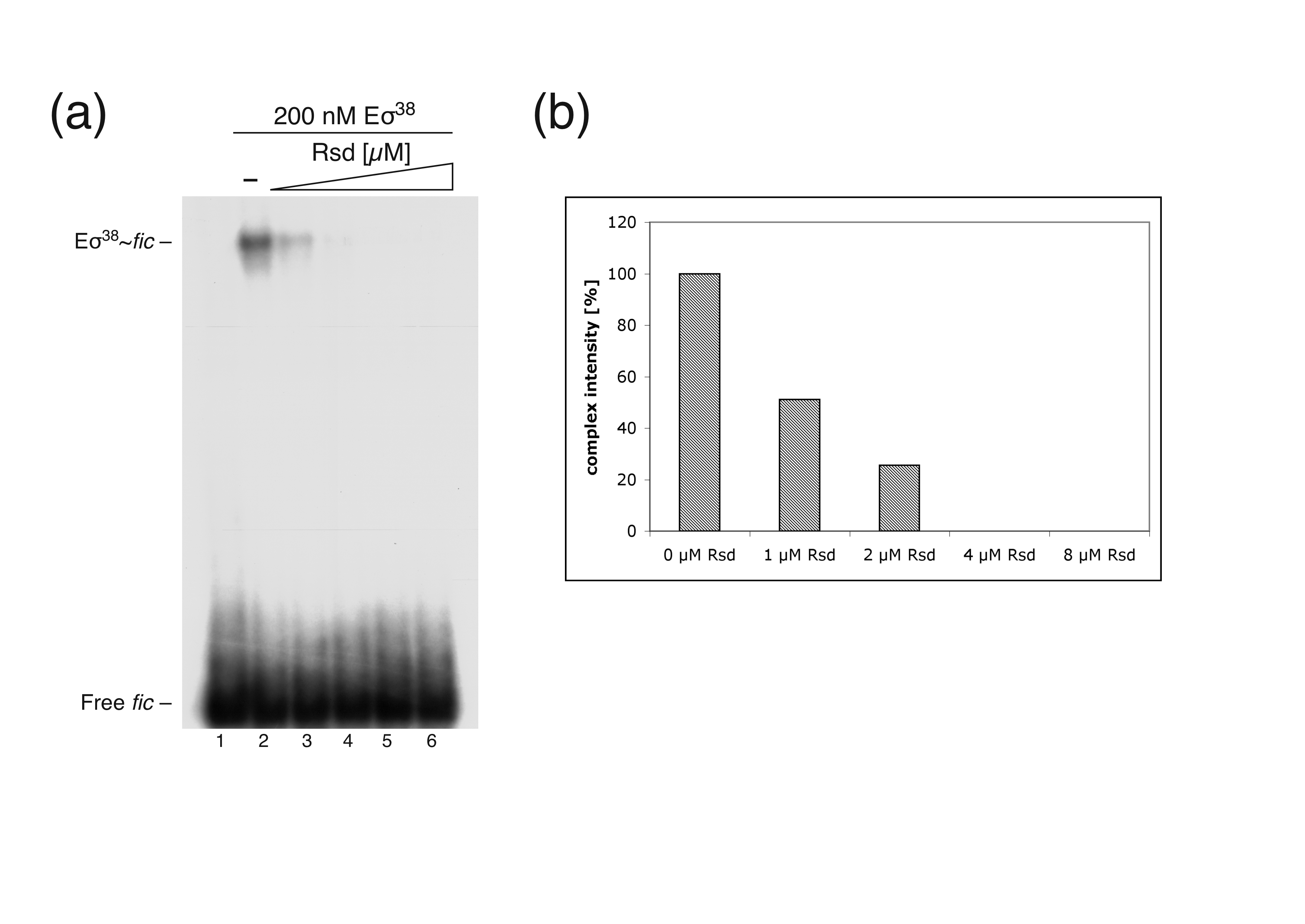

Supplement: Figure S3 — Effect of Rsd on Eσ38∼ fic promoter complex formation. (a) Binding of the RNA polymerase Eσ38 holoenzyme (200 nM) to a DNA fragment harbouring the σ38-dependent fic promoter was analyzed by gel retardation. Complex formation was challenged by increasing concentrations of Rsd. In lane 1 the free DNA is shown. Lane 2 represents the complex in the absence of Rsd. In lane 3 to 6 increasing Rsd concentrations of 1 µM (lane 3), 2 µM (lane 4), 4 µM (lane 5) and 8 µM (lane 6) were present. (b) Diagram showing the quantitative evaluation of the data from (a) indicating the remaining amounts of complex as a function of the Rsd concentration. (TIFF) [file pone.0019235.s003.tif]

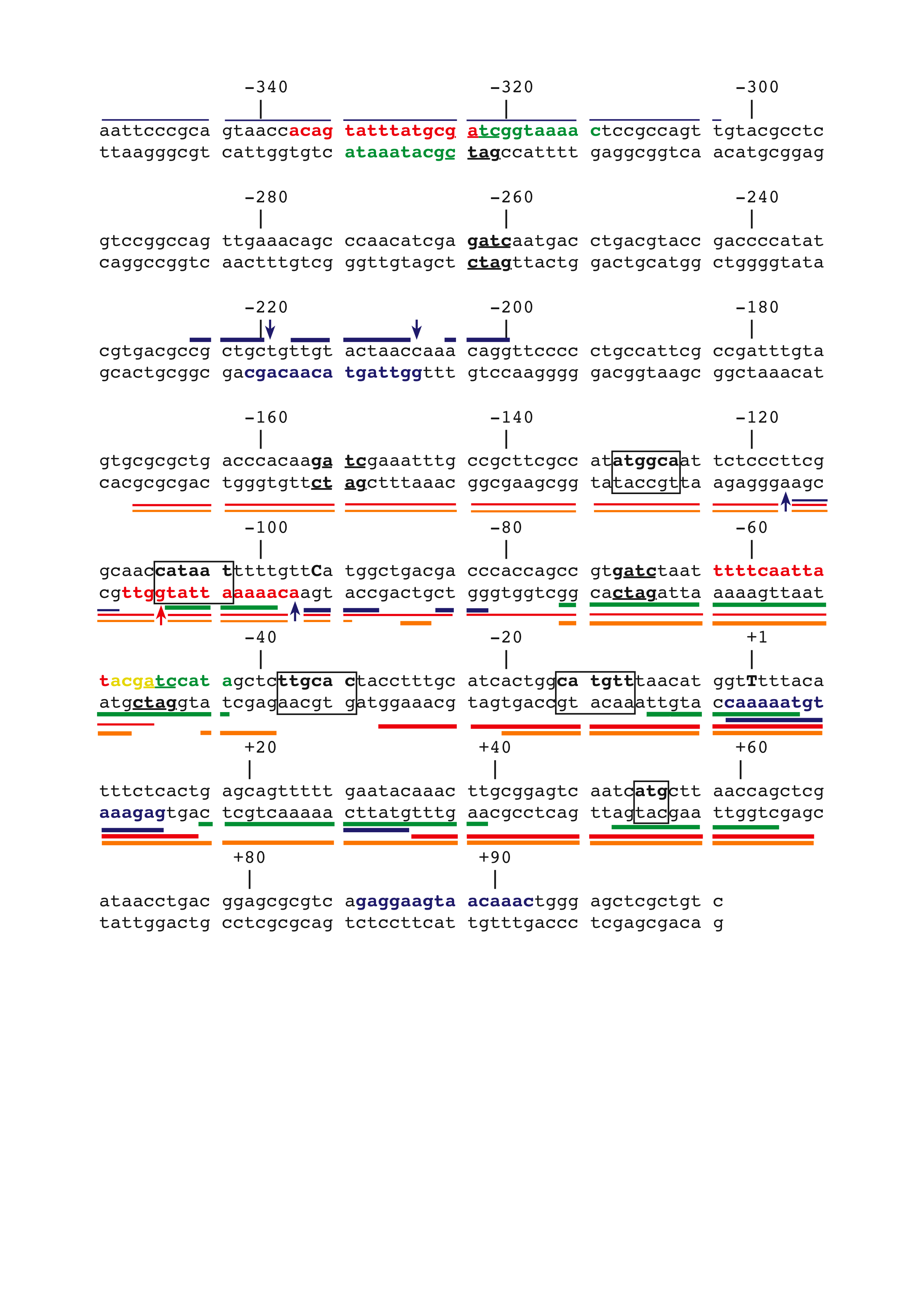

Supplement: Figure S4 — Sequence of the rsd promoter region. The sequence given corresponds to the rsd-up fragment presented in Figure 5a. Numbers indicate sequence positions relative to the rsd P2 transcription start site. Promoter core elements (−10 and −35 regions) are boxed and highlighted with bold letters. Transcription start sites of the rsd P1 and P2 promoters are marked by bold-type capital letters. The Rsd translation initiation codon is shown in bold-type and boxed. The five GATC sites are bold-type and underlined. NAP binding sites and the respective colour code are taken from Figure 6. The thickness of the lines represents high or low affinity of the respective NAPs. Hyperreactive sites are indicated by arrows. Sequence positions matching the known consensus sites for H-NS, LRP and FIS [39], [40] are highlighted in bold-type with the colour given in the key of Figure 6. Overlapping sites between H-NS and LRP are shown in yellow colour. (TIFF) [file pone.0019235.s004.tif]

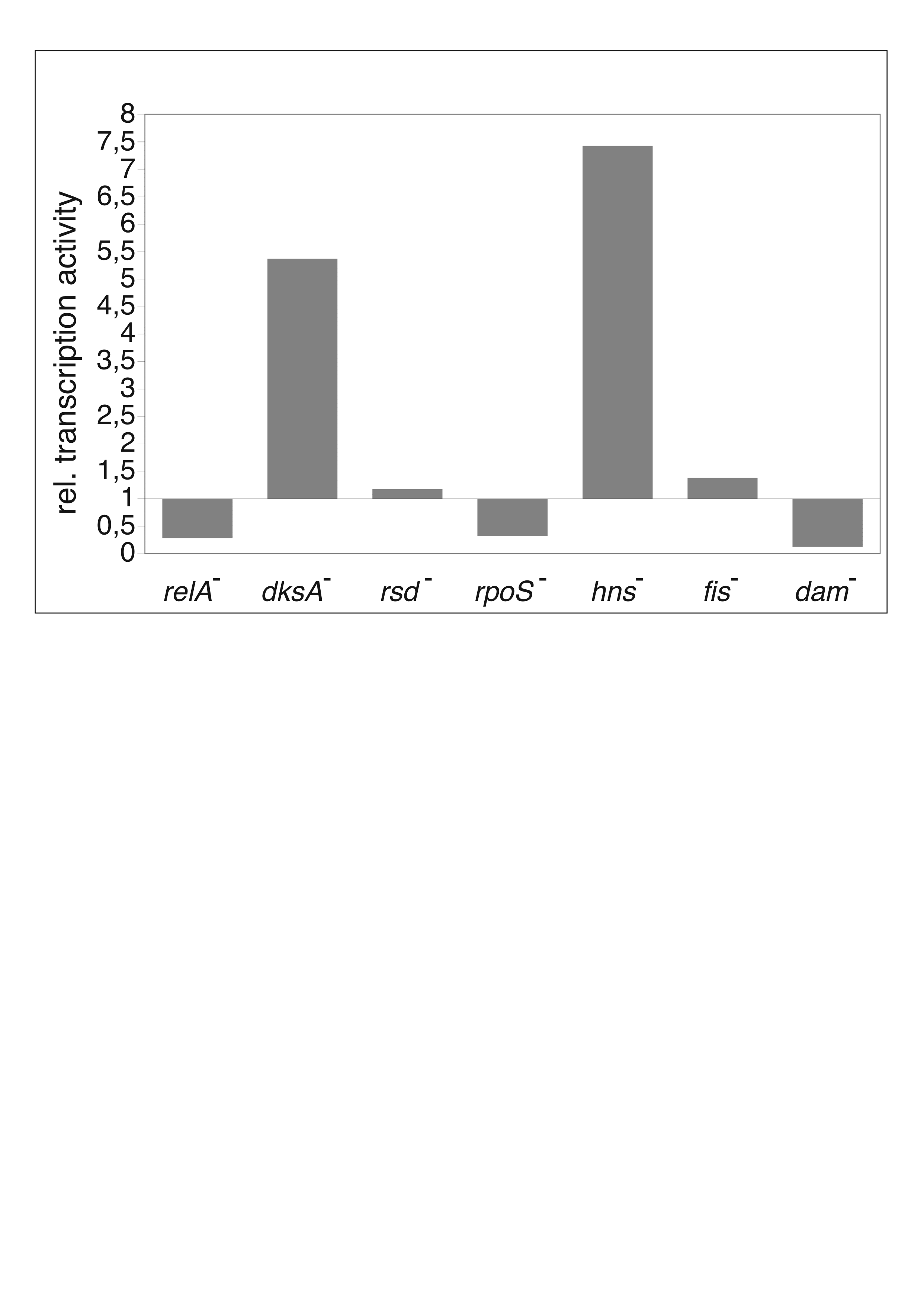

Supplement: Figure S5 — rsd P1 promoter activity in different strain background during stationary phase of growth. For the rsd P1 promoter notable amounts of transcript were only obtained during stationary phase of growth. The diagram depicts the relative amounts of rsd P1-derived transcripts normalized to the RNA 1. Compared are the transcripts from strains with defects in relA, dksA, rsd, rpoS, hns, fis and dam relative to the transcripts from the respective wild-type strains, normalized to 1. Shown is one representative experiment out of two to three with similar results. (TIFF) [file pone.0019235.s005.tif]
