# Supplementary material for: The E. coli Anti-Sigma Factor Rsd: Studies on the Specificity and Regulation of Its Expression
Source: PLoS One. 2011 May 6;6(5):e19235. doi: 10.1371/journal.pone.0019235 (PMC3089606; doi:10.1371/journal.pone.0019235)
Supplement: Table S1 — Bacterial strains and plasmids used in this study. (DOC) [file pone.0019235.s006.doc]

**Supplementary Table S1**

**Bacterial strains and plasmids used in this study**

| **Strain or plasmid** | **Relevant characteristics** | **Reference** |
| --- | --- | --- |
| **Strains** | | |
| BL21DE3 pLysS | F^-^, *omp*T, *hsd* S_B_ (r_B_^-^m_B_^-^), *dcm*, *gal*, (DE3), pLysS, Cm^r^ | [S2, S3] |
| CF9239 | MG1655 (F^-^) ∆*dksA:: kan^r^* | A gift from M. Cashel  [S4] |
| CJD1124 | GM37; *stpA^-^, tet^r^* | [S5] |
| CP78 | F^+^, *thi^-^, leu^-^, thr^-^, his^-^, arg^-^, mal^-^, xyl^-^, ara^-^, gal^-^, str^s^, rel A^+^* | [S6] |
| CP79 | CP78; *rel A^-^* | [S6] |
| CSH50 | F^-^; ara, ∆*(pro-lac), str A, thi* | [S7] |
| CSH50fis::kan | CSH50; *fis*^-^ | [S8] |
| GM37 | see CJD 1124 | [S5] |
| HB 101 | F^-^, *pro* A2, *rec*A13, *ara*-14, *lac*Y1, *gal*K2, *xyl*-5, *mtl*-1, *rps* L20 (Str^r^) | Laboratory collection [S9] |
| JM110 | F’, *tra* D36, *lac I*^q^, *lac* Z∆M15, *pro AB*/*rps L* (Str^r^), *thr, leu, thi, lac Y, gal K, gal T, ara, fhu A, tsx, dam, dcm, sup E44,* ∆(*lac-pro AB*) | Laboratory collection [S10] |
| MC4100 | F’, *araD*139, ∆ (*argF-lac*) U169, *deoC*1, *flb*5301, *relA*1, *rpsL*150, *ptsF*25, *rbsR* | Laboratory collection [S11] |
| MG1655 | *E. coli* K12 wild type | Laboratory collection  [S12] |
| MG1655*rsd*::kan | MG1655 *rsd^-^*, Kan^r^ | [29] |
| PD32 | MC4100, *hns*-206::Ap^r^ | [S13] |
| RH90 | MC4100, *rpoS*359:: Tn10; | [S14] |
| XL-1 | *rec*A1, *lac*^-^, *end* A1 *gyr* A96, *thi*, *hsd* R17, *sup* E44, *rel* A1, [F^-^: *pro* AB, *lac*^iq^, *lac*Z ΔM15, Tn 10] | Stratagene |
| **Plasmids** | | |
| pUC18-Rsd | pUC18-T7 derivative with the *rsd* gene | This work |
| pUC18-rsd-up | pUC18 derivative with the *rsd* promoter including a 230 bp long upstream regulatory region | This work |
| pUC18-bolA | pUC18 derivative with *E. coli bolA* promoter | Gift of B. Reckendrees |
| pUC18-bolA1 | pUC18 derivative with *E. coli bolA* promoter without upstream regulatory region | Gift of B. Reckendrees |
| pSH666-1 | pKK223-3 derivative with the *rrnB* P1 and *bolA* promoters both terminated by the *rrnB* T1 T2 terminater | Gift of P. Schoengraf |
| pKK223-3 | Expression vector with tac promoter | [S15] |
| p*rsd*-up-cat | pKK232-8 derivative with the 499 bp *rsd* promoter upstream region from pUC18-rsd-up | This work |
