# Supplementary material for: The E. coli Anti-Sigma Factor Rsd: Studies on the Specificity and Regulation of Its Expression
Source: PLoS One. 2011 May 6;6(5):e19235. doi: 10.1371/journal.pone.0019235 (PMC3089606; doi:10.1371/journal.pone.0019235)
Supplement: Table S2 — Quantitative evaluation of RNA polymerase complexes formed with methylated and non-methylated rsd promoter DNA. (DOC) [file pone.0019235.s007.doc]

**Supplementary Table S2**

**Quantitative evaluation of RNA polymerase complexes formed with methylated and non-methylated *rsd* promoter DNA.**

| **Non-methylated fragment** | | | | | | |
| --- | --- | --- | --- | --- | --- | --- |
|  | **Eσ^70^** | | **Eσ^38^** | | **Eσ^70/38^** | |
| **Rsd** | **–** | **+** | **–** | **+** | **–** | **+** |
| ***rsd*~P2 complex** | 20% | 12% | < 1% | < 1% | 16% | 8% |
| ***rsd*~P1 complex** | n.d. | n.d. | 5% | 6% | n.d. | 2% |
| **Methylated fragment** | | | | | | |
|  | **Eσ^70^** | | **Eσ^38^** | | **Eσ^70/38^** | |
| **Rsd** | **–** | **+** | **–** | **+** | **–** | **+** |
| ***rsd*~P2 complex** | 49% | 30% | 8% | ~ 1% | 47% | 10% |
| ***rsd*~P1 complex** | n.d. | n.d. | 22% | 27% | 4% | 19% |

Numbers were derived from a non-saturated autoradiogram of the experiment shown in Figure 5.
