# Supplementary material for: The E. coli Anti-Sigma Factor Rsd: Studies on the Specificity and Regulation of Its Expression
Source: PLoS One. 2011 May 6;6(5):e19235. doi: 10.1371/journal.pone.0019235 (PMC3089606; doi:10.1371/journal.pone.0019235)
Supplement: Text S1 — Supplementary References. (DOC) [file pone.0019235.s008.doc]

**Text S1**

**Supplementary References**

S1. Schröder O, Wagner R (2000) The bacterial DNA-binding protein H-NS represses ribosomal RNA transcription by trapping RNA polymerase in the initiation complex. J Mol Biol 298: 737-748.

S2. Studier FW, Moffatt BA (1986) Use of bacteriophage T7 RNA polymerase to direct selective high-level expression of cloned genes. J Mol Biol 189: 113-130.

S3. Davanloo P, Rosenberg AH, Dunn JJ, Studier FW (1984) Cloning and expression of the gene for bacteriophage T7 RNA polymerase. Proc Natl Acad Sci U S A 81: 2035-2039.

S4. Kang PJ, Craig EA (1990) Identification and characterization of a new *Escherichia coli* gene that is a dosage-dependent suppressor of a *dnaK* deletion mutation. J Bacteriol 172: 2055-2064.

S5. Free A, Williams RM, Dorman CJ (1998) The StpA protein functions as a molecular adapter to mediate repression of the *bgl* operon by truncated H-NS in *Escherichia coli*. J Bacteriol 180: 994-997.

S6. Fiil N, Friesen JD (1968) Isolation of relaxed mutants of *E. coli*. J Bacteriol 95: 729-731.

S7. Miller JH (1972) Experiments in Molecular Genetics. New York: Cold Spring Harbor Laboratory Press.

S8. Koch C, Vandekerckhove J, Kahmann R (1988) *Escherichia coli* host factor for site-specific DNA inversion: cloning and characterization of the *fis* gene. Proc Natl Acad Sci USA 85: 4237-4241.

S9. Boyer HW, Roulland-Dussoix D (1969) A complementation analysis of the restriction and modification of DNA in *Escherichia coli*. J Mol Biol 41: 459-472.

S10. Yanisch-Peron C, Vieira J, Messing J (1985) Improved M13 mp18 pUC19 vectors. Gene 33: 103-119.

S11. Casadaban MJ (1976) Transposition and fusion of *lac* genes to selected promoters in *Escherichia coli* using bacteriophage lambda and Mu. J Mol Biol 104: 541-555.

S12. Blattner FR, Plunkett III G, Bloch CA, Perna NT, Burland V, et al. (1997) The complete genome sequence of *Escherichia coli* K-12. Science 277: 1453-1462.

S13. Dersch P, Schmidt K, Bremer E (1993) Synthesis of the *Escherichia coli* K-12 nucleoid-associated DNA-binding protein H-NS is subjected to growth-phase control and autoregulation. Mol Microbiol 8: 875-889.

S14. Lange R, Hengge-Aronis R (1991) Growth phase-regulated expression of *bolA* and morphology of stationary- phase *Escherichia coli* cells are controlled by the novel sigma factor sigma S. J Bacteriol 173: 4474-4481.

S15. Brosius J, Lupski JR (1987) Plasmids for the selection and analysis of prokaryotic promoters. Methods Enzymol 153: 54-68.
